# Genome-wide mRNA profiling in urinary extracellular vesicles reveals stress gene signature for diabetic kidney disease

**DOI:** 10.1016/j.isci.2023.106686

**Published:** 2023-04-18

**Authors:** Om Prakash Dwivedi, Karina Barreiro, Annemari Käräjämäki, Erkka Valo, Anil K. Giri, Rashmi B. Prasad, Rishi Das Roy, Lena M. Thorn, Antti Rannikko, Harry Holthöfer, Kim M. Gooding, Steven Sourbron, Denis Delic, Maria F. Gomez, iBEAt, Per-Henrik Groop, Tiinamaija Tuomi, Carol Forsblom, Leif Groop, Maija Puhka

**Affiliations:** 1 Institute for Molecular Medicine Finland FIMM, HiLIFE, University of Helsinki, Helsinki, Finland; 2 Institute for Molecular Medicine Finland FIMM, EV and HiPrep Core, University of Helsinki, Helsinki, Finland; 3 Department of Primary Health Care, Vaasa Central Hospital, Hietalahdenkatu 2-4, 65130 Vaasa, Finland; 4 Diabetes Center, Vaasa Health Care Center, Sepänkyläntie 14-16, 65100 Vaasa, Finland; 5 Folkhälsan Institute of Genetics, Folkhälsan Research Center, Helsinki, Finland; 6 Department of Nephrology, University of Helsinki and Helsinki University Hospital, Helsinki, Finland; 7 Research Program for Clinical and Molecular Metabolism, Faculty of Medicine, University of Helsinki, Helsinki, Finland; 8 Foundation for the Finnish Cancer Institute (FCI), Tukholmankatu 8, 00290 Helsinki, Finland; 9 iCAN Digital Precision Cancer Medicine Flagship, University of Helsinki, Helsinki, Finland; 10 HiLIFE-Helsinki Institute of Life Science, University of Helsinki, Helsinki, Finland; 11 Lund University Diabetes Centre, Department of Clinical Sciences, Lund University, SE 214 28 Malmö, Sweden; 12 Institute of Biotechnology, University of Helsinki, Helsinki, Finland; 13 Research Program in Systems Oncology, Faculty of Medicine, 00014 University of Helsinki, Helsinki, Finland; 14 Department of Urology, 00014 University of Helsinki, and Helsinki University Hospital, 00100 Helsinki, Finland; 15 Department of Medicine, University Medical Center, Hamburg-Eppendorf, Hamburg, Germany; 16 Diabetes and Vascular Research Centre, National Institute for Health Research Exeter Clinical Research Facility, University of Exeter Medical School, Exeter, UK; 17 Department of Imaging, Infection, Immunity and Cardiovascular Disease, University of Sheffield, Sheffield, UK; 18 Boehringer Ingelheim Pharma GmbH & Co. KG, Biberach, Germany; 19 Fifth Department of Medicine, Nephrology/Endocrinology/Rheumatology/Pneumology, University Medical Centre Mannheim, University of Heidelberg, Heidelberg, Germany; 20 Department of Diabetes, Central Clinical School Monash University, Melbourne, VIC, Australia; 21 Endocrinology, Abdominal Centre, Helsinki University Hospital, Helsinki, Finland

**Keywords:** Medicine, Clinical finding, Disease, Specimen, Biopsy sample

## Abstract

Urinary extracellular vesicles (uEV) are a largely unexplored source of kidney-derived mRNAs with potential to serve as a liquid kidney biopsy. We assessed ∼200 uEV mRNA samples from clinical studies by genome-wide sequencing to discover mechanisms and candidate biomarkers of diabetic kidney disease (DKD) in Type 1 diabetes (T1D) with replication in Type 1 and 2 diabetes. Sequencing reproducibly showed >10,000 mRNAs with similarity to kidney transcriptome. T1D DKD groups showed 13 upregulated genes prevalently expressed in proximal tubules, correlated with hyperglycemia and involved in cellular/oxidative stress homeostasis. We used six of them (*GPX3*, *NOX4*, *MSRB*, *MSRA*, *HRSP12,* and *CRYAB*) to construct a transcriptional “stress score” that reflected long-term decline of kidney function and could even identify normoalbuminuric individuals showing early decline. We thus provide workflow and web resource for studying uEV transcriptomes in clinical urine samples and stress-linked DKD markers as potential early non-invasive biomarkers or drug targets.

## Highlights

• NGS reveals robust expression of >10,000 mRNAs in clinical uEV samples
• uEV transcriptome enriches kidney mRNAs and shows global similarity with the kidney
• In DKD, uEV express elevated levels of cellular/oxidative stress-linked genes
• uEV stress score associates with long-term decline of eGFR in early and late DKD

## Graphical abstract

**Figure:**
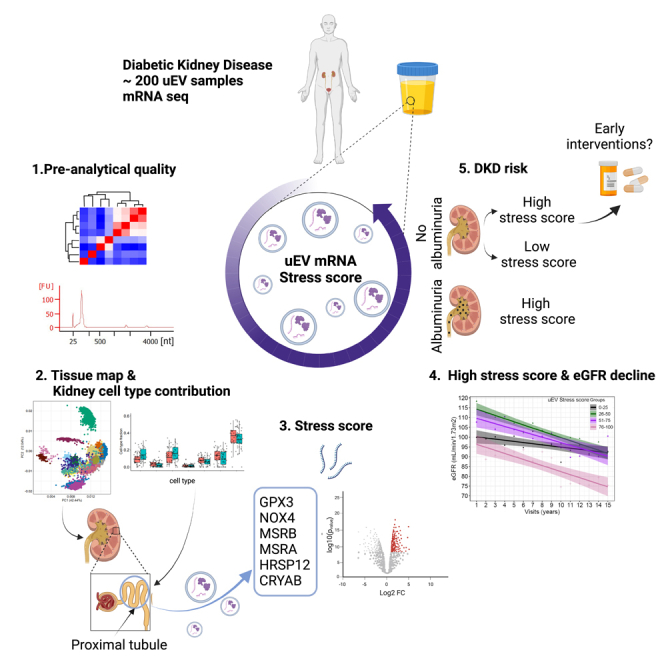

## Introduction

Diabetes is a multifactorial disease with diverse complications causing a global health burden and >1.3 million annual deaths.^1^ One of the most severe complications is diabetic kidney disease (DKD)—a leading cause of end-stage renal disease.^2^ Kidney biopsy is considered as the gold standard to observe the DKD-linked structural changes and progression stages.^3^ However, in practice, the diagnosis is usually based on albuminuria and decline in kidney function measured as glomerular filtration rate.^4^^,^^5^ Despite their wide clinical applications, these traditional markers perform poorly in detecting early stages of DKD, for example hyperfiltration and in prognosticating disease progression.^6^

Transcriptomics of the kidney tissue has revealed association of kidney mRNA markers with DKD related structural changes like fibrosis^7^^,^^8^ and led to development of biomarkers including epidermal growth factor as a chronic kidney disease marker.^9^ However, the risks associated with kidney biopsies, in particular at later stages of DKD, have limited kidney transcriptome-based approaches in DKD precision medicine.^10^ This has turned the interest to extracellular RNAs in urine that might mirror the pathological changes of kidney transcriptome and provide an alternative non-invasive way for diagnosing, prognostication, and monitoring DKD.^11^

Extracellular vesicles (EV) are lipid bilayer contained particles secreted by cells in varying sizes (∼30–1000 nm). They carry active biomolecules (proteins, lipids, metabolites, DNA, and RNA) and contribute to many biological processes in health and disease e.g., through their role in cell-to-cell signaling.^12^^,^^13^^,^^14^ EV are found in all body fluids, where their membrane helps to protect the cargo from proteases and RNases.^15^ There has been a global effort to map RNAs in EV from various human body fluids;^16^^,^^17^ however, the predominant focus has been on small RNAs.^18^^,^^19^^,^^20^ Comprehensive characterization of long RNAs (like mRNAs) in EV from clinical samples is lacking because of technical challenges posed by low RNA quantities and non-standardized workflows. Standardization of protocols for large-scale clinical studies is currently one of the main objectives in the urine EV (uEV) field.^21^^,^^22^^,^^23^

In this study, we utilized low input mRNA sequencing (mRNAseq) for uEV and analyzed uEV mRNAs in ∼200 samples from five clinical studies including participants of three different type 1 diabetes (T1D) and type 2 diabetes (T2D) studies presenting varying stages of DKD (normo, micro, or macroalbuminuria). We show 1) technical quality and reproducibility of the uEV mRNAseq pipeline and feasibility in clinical setting by combining cohorts with different urine collection protocols, 2) comprehensive characterization of uEV mRNA transcriptomes suggesting similarity with kidney, and 3) identification of DKD candidate marker genes involved in cellular stress responses and linking them with long-term kidney function decline.

## Results

### Study design

**Figure 1:**
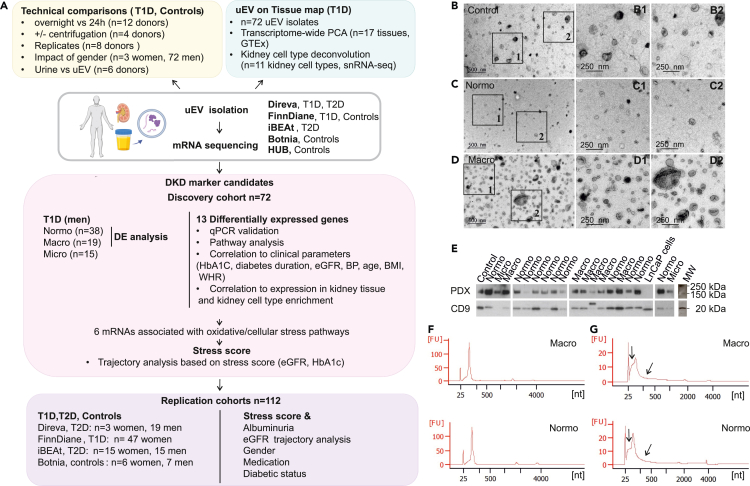
Study design and Urinary extracellular vesicles (uEV) quality control (A) Study design for the next generation sequencing of uEV mRNAs to assess urine collections, quality and reproducibility (technical comparisons), origins of uEV transcripts (uEV on Tissue map), and discovery of candidate non-invasive markers for diabetic kidney disease (DKD marker candidates). (B–D) Transmission electron micrographs showing uEV of typical morphology and variable sizes; filamentous structures compatible with Tamm-Horsfall protein filaments are visible in the in-set B1. (E) Western blotting showing the presence of uEV-enriched markers CD9 and PDX in uEV preparations. (F) Representative electropherograms of RNA isolated from uEV and analyzed with bioanalyzer using Agilent Pico kit. An RNA peak between 25 and 200 nt was detected in all samples and with this characteristic shape passed quality control. (G) Examples showing signs of nucleic acid degradation (arrows) in uEV RNA samples and not passing quality control. 24 h (24h); albumin excretion rate (AER); blood pressure (BP); body-mass index (BMI); estimated glomerular filtration rate (eGFR); waist-to-hip ratio (WHR); Genotype-Tissue Expression (GTEx) project; glycated hemoglobin (HbA1_C_); Lymph Node Carcinoma of the prostate (LnCaP), macroalbuminuria (Macro); microalbuminuria (Micro); non-diabetic control (Control); normoalbuminuria (Normo); messenger RNA sequencing (mRNAseq); Podocalyxin (PDX), type 1 or type 2 diabetes (T1D, T2D); urinary extracellular vesicles (uEV); principal component analysis (PCA); single-nucleus RNA sequencing (snRNA-seq).

The first part of our uEV mRNAseq study included >100 samples and focused on three main aims: 1) to assess the feasibility, quality, and reproducibility of low input uEV mRNAseq for different types of clinical urine collections, 2) to assess whether uEV could capture gene expression signatures of the kidney or other tissues, and 3) to discover novel candidate genes for DKD focusing on individuals with T1D (Figure 1A). The tested technical variables were: 1) overnight (ON) vs. 24-h urine collection, 2) urine samples processed with or without centrifugation prior to freezing, and 3) technical replicates of urine passing through the whole pipeline from uEV isolation to mRNAseq at 1–5 months intervals, 4) impact of gender, and 5) impact and criteria of RNA quality demonstrated by comparing urine vs. uEV (Figure 1A, technical comparisons).

Additionally, we compared the mRNA expression profiles of uEV with various tissues in the genotype-tissue expression (GTEx) project^24^ (Figure 1A, tissue map) and also estimated the kidney cell type proportions based on deconvolution of uEV mRNAs using kidney single-nucleus sequencing database as a ref.^25^

To find novel DKD candidate genes, our discovery phase study included global mRNAseq for uEV samples from 72 individuals collected from two T1D cohorts (FinnDiane, Finnish Diabetic Nephropathy Study, and DIREVA, Diabetes Registry in Vaasa) with different urine collection types (24-h and ON urine collection, respectively) (Figure 1A). The included donors/samples differed regarding the degree of albuminuria (normoalbuminuria, microalbuminuria, and macroalbuminuria) as well as estimated glomerular filtration rate (eGFR), HbA_1c_ (glycated hemoglobin A1c), and diabetes duration (Tables S1, S2 and S4). We also utilized the long-term (13–15 years) clinical laboratory data (eGFR, HbA_1c_) for association analysis. The expression of the candidate genes identified in the discovery T1D study were further analyzed again in >100 uEV samples including three different replication cohorts of 1) women with T1D and 2) two different T2D cohorts with varying kidney function (Table S3). As the discovery phase included only men to reduce heterogeneity (e.g., due to differences in the genitourinary system), we assessed the association of candidate gene signature with kidney dysfunction with primary focus on uEV samples of women’s T1D cohort in replication phase.

### Quality control of uEV

Urinary EV isolates obtained by ultracentrifugation from the different study groups produced overall a good uEV and RNA quality, as shown by electron microscopy (EM), Western blotting, and EV RNA profiling by bioanalyzer pico assay (Figure 1) as well as by nanoparticle tracking analysis (NTA, Figure S1). We detected typical round EV showing different staining intensities, sizes, and morphologies by EM (Figures 1B–1D). The isolates contained also some Tamm-Horsfall protein filaments, but not in excessive quantities. The EV were roughly up to 1 μm in diameter, although the main population consisted of smaller (exosomal range) sizes (30–150 nm). NTA indicated a similar size range of particles (Figure S1A). Western blotting showed variable quantities of two uEV-enriched proteins, CD9, and podocalyxin, in the study samples (Figure 1E). Despite the variable EV marker quantities, sufficient amounts of total RNA could be extracted from all EV samples. We have already previously found the uEV quality from the ultracentrifugation protocols to be good after a thorough characterization of subsets of samples from the FinnDiane, DIREVA, and HUB (Helsinki Urological Biobank) study cohorts mainly including men, individuals with T1D and controls.^17^^,^^22^^,^^23^

Bioanalyzer showed typical RNA profiles for the uEV from all study groups: a relatively large amount of small RNA and no or minor peaks of rRNA (Figure 1F). However, we observed that the RNA profiles from up to 27% of the uEV from women showed some signs of degradation in contrast to only 1% of the uEV from men in replication diabetes cohorts. We defined these signs as a “shoulder” of the main small RNA peak toward the marker peak or a slight tail toward bigger RNA sizes (Figure 1G). Similar profiles are characteristic to e.g., formalin-fixed paraffin-embedded samples known to contain degraded (cellular) RNA (Illumina technical document^26^) or total urine RNA samples (Figure S1). To look deeper into these quality criteria, we first assessed pairs of urine RNA containing and matching uEV RNA lacking the degradation signs (examples in Figure S1B). Messenger RNA sequencing of six such pairs of urine and their matching uEV (HUB study, n = 5 male, 1 female) revealed that the worse bioanalyzer profiles from urines associated with significantly reduced mapped read numbers as compared with the uEV (>3-fold, p < 0.001, Figure S1C). A similar difference was obtained when comparing the mapped reads between uEV samples with good and bad RNA profiles from a subset of T1D women’s cohort (>2-fold, p < 0.01, Figure S1D). We additionally noted that ∼27% of this subset contained >5% of intergenic reads (10 kb upstream or downstream of coding regions) that may stem from DNA. Majority (85%) of these DNA containing samples showed also signs of RNA degradation by bioanalyzer. Thus, to avoid problems caused by degraded RNA or DNA, we excluded samples based on bioanalyzer and >5% of intergenic reads (when information was available). The diminished number of mapped reads was evident when comparing all excluded vs. included samples of the T1D women's subset (>2-fold, p < 0.01, Figure S1D).

### Quality, robustness, and reproducibility of uEV mRNA profiling by next generation sequencing

We performed next generation sequencing **(**NGS) of uEV mRNAs using methods accepting low RNA input. For discovery, we used both T1D cohorts (total n = 72 men, 24-h or ON urine collection) (Figure 1) that produced a median (IQR) of 11.5 million (7.4–16.3) raw sequencing reads (Figure S2). Most raw sequencing reads (median% [IQR], 90.4% [87.6–91.9]) mapped uniquely to the human genome with <7% of unmapped or multi-mapped reads (4.6% [3.7–5.9] and 4.7% [3.7–6.5], respectively) (Figure S2A). Furthermore, as expected, most uniquely mapped reads aligned to exonic genomic regions (coding sequence (CDS): 73.6% [72.3–74.6], 3′ untranslated region (UTR): 14.5% [13.7–15.7] for exons and 5′UTR:11.6% [10.9–12.8]), and <0.1% mapped to intronic or intergenic regions (10 kb upstream or downstream of coding regions) (Figure S2B). We detected robust expression of 10,596 protein coding genes in uEV from the T1D discovery cohorts that were expressed in >90% of samples (with count ≥1, Figure S2E).

**Figure 2:**
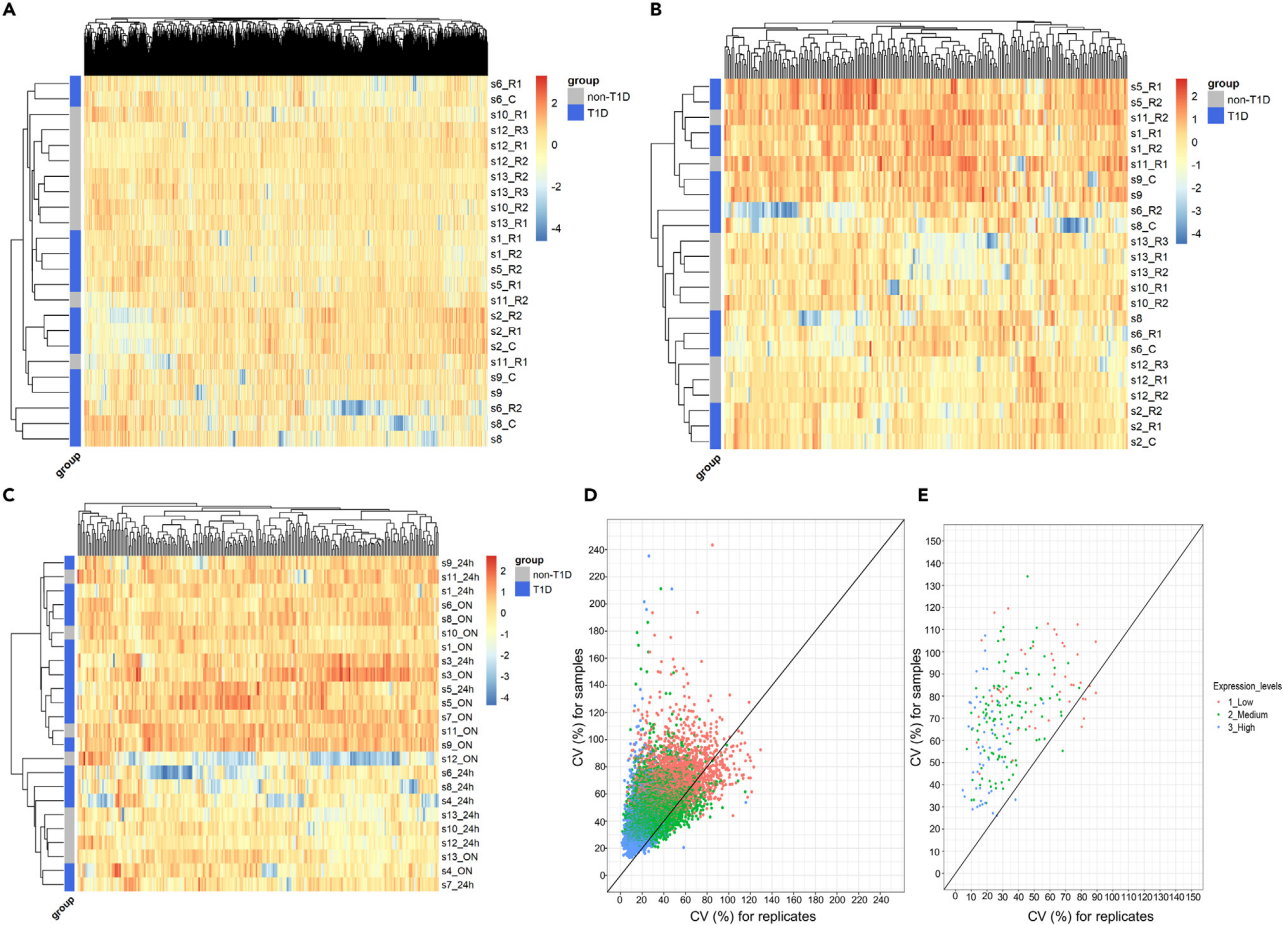
Sequencing of uEV mRNAs showed consistent gene expression distribution and reproducibility across different technical comparisons (A and B) Clustering of all technical replicates (from diabetic and non-diabetic individuals) using uEV expression levels across A-all commonly expressed genes (n = 10,449) and B- kidney enriched genes (n = 233). Technical replicates included 1) aliquots of individual samples (24h urine collection) processed through the uEV mRNA sequencing pipeline twice (R1 and R2, n = 6 donors) or thrice (R1, R2 and R3, n = 2) at different time points and 2) urine samples (24h collection) processed with (_C) and without centrifugation before freezing (n = 4). (C) Clustering based on expression levels of kidney enriched genes in uEV for all technical samples from ON vs. 24h urine collections. Samples from individual donors were collected from the same day (n = 12 donors). Samples S1-S9 were from individuals with T1D and S10-S13 from non-diabetic individuals. The gene expression values (log2CPM) were inverse normally transformed and converted to *Z* score before analysis for A-C. Clustering analysis (A to C) was performed using Euclidean distance-based method. (D and E) Coefficient of variation (CV%) comparing technical replicates (X axis, n = 2 unique control samples with triplicate measurements) with all T1D samples of discovery cohort (Y axis, n = 72) using all uEV genes (D, n = 9,542- genes expressed in 3 replicates and present in T1D discovery cohort set) and kidney enriched genes (E, n = 211- detected in 3 replicates). The color coding represents the average expression level (low expression = 0-25 percentile, n = 2390 or 47 genes; medium expression = 26-74 percentile, n = 4765 or 113 genes; high expression = 75-100 percentile, n = 2387 or 51). Counts per million (CPM); replicate (R); type 1 diabetes (T1D); urinary extracellular vesicles (uEV).

We further tested the robustness of uEV mRNA profiles using pairs of samples for three different technical variables, 1) urine collection protocol: 24-h vs. ON, 2) effect of centrifuging the urine samples prior to freezing (±centrifugation), and 3) reproducibility by processing aliquoted urine samples through the whole uEV mRNAseq pipeline— from uEV isolation to sequencing— at different time points (replicates) (Figure 1 and Table S5). In these three technical comparisons, similar to the main T1D study cohorts, we observed (median-IQR) 11.5 (8.6–13.5) million raw sequencing reads that were mostly [91.6% (88.2–93.9)] uniquely mapped to human genome, particularly to the exonic gene regions (Figures S3A and S3B). We detected the expression of 10,449 protein coding genes (with count ≥1) present in ≥90% of samples used for the three technical comparisons. The read count data for these commonly expressed genes (10,449) showed clustering (based on correlation) for most technical replicates (Figure S3C). We performed Euclidean distance-based clustering of expression profiles for all 12 sample pairs/triplicates using all common detected protein coding genes (Figure 2A, n = 10,449) or “kidney-enriched genes” (Figure 2B, n = 233 present in uEV)—a subset of protein coding genes known to be enriched in kidney (see STAR Methods) and expressed in our technical uEV sample sets. Pairs or triplicates of replicate urine samples and those with or without centrifugation showed high correlation (r = 0.81 to 0.95) and most of these technical pairs clustered together (9 out of 12) both when all or only kidney enriched genes were considered regardless of their donor’s diabetic and non-diabetic status (Figures 2A and 2B). All 24-h and ON pairs did not cluster together, but no systematic difference was observed for the urine sample pairs either from T1D individuals or non-diabetic individuals regarding all commonly expressed genes (Figure S4) or kidney-enriched genes (Figure 2C). When comparing discovery population samples with technical triplicates, we observed 1.6 to 2-fold higher variability (coefficient of variation -CV %) in all expressed genes (49% vs. 30%) or kidney-enriched genes (71% vs. 34%) (Figures 2D and 2E) in population samples. Most variability in all samples was mainly contributed by low-expressed genes (Figures 2D and 2E).

Finally, we also tested any sex driven global differences in uEV mRNA profiles by clustering of three samples from women with all the men from the T1D cohorts (as mentioned in Figure 1A, n = 72). Men and women clustered mainly separately based on sex chromosome genes and all robustly expressed uEV genes (n = 10,596 genes), but sexes did not separate based on kidney-enriched genes (n = 247 present in uEV out of 413, seeSTAR Methods) (Figure S4).

Together, these results showed the expression of >10,000 protein coding genes in uEV from different clinical cohorts. It is noteworthy that this is the first study that successfully combined a relatively large number of samples from different urine collections highlighting the comparability and reproducibility of the uEV mRNA profile and the used low input technologies.

### Similarity between uEV and kidney transcriptomes and kidney cell type deconvolution analysis

The uEV could be derived from several tissues—thus, to study the origin of the uEV mRNAs, we compared the uEV transcriptome to individual organ-specific gene expression signatures. We obtained sets of known “kidney-enriched genes” (413 genes with higher expression in kidney than in other tissues, see STAR Methods) and “kidney-depleted genes” (4,631 genes not detected in kidney) from the human protein atlas database (https://www.proteinatlas.org/humanproteome/tissue/kidney) and checked their expression in T1D uEV (with the total of n = 10,596 genes). We detected the expression of 59.8% (247 out of 413) of kidney-enriched genes compared to only 4.3% (200 out of 4,631) of kidney-depleted genes in the T1D uEV. We further looked at more “specific kidney-enriched genes”- a subset of kidney-enriched genes that are reported to be detected in only few human tissues other than kidney (see STAR Methods) and detected the expression of 40.5% of such genes (77 out of 190) (Figure S5A).

**Figure 3:**
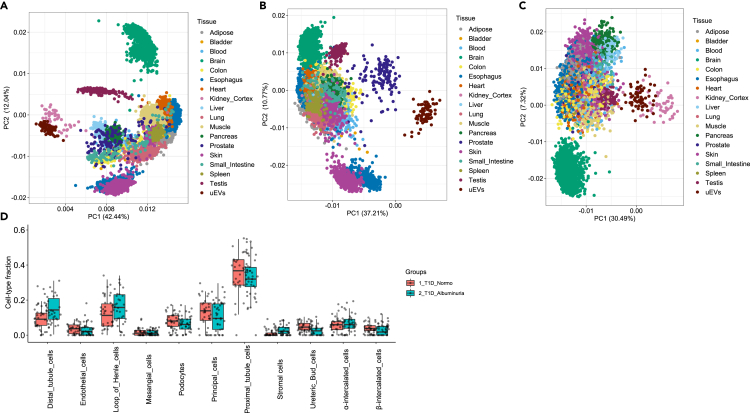
Genome-wide mRNA profile of uEV showed similarity with the kidney mRNA profile and deconvoluted kidney cell types (A–C) PC analysis using gene expression levels (log2CPM) in uEV from the T1D cohorts (n = 72), and the respective gene expression levels (log2CPM) in 17 human tissues obtained from the GTEx reference database (tissues from men only; Adipose = 692, Bladder = 9, Blood = 426, Brain = 1612, Colon = 475, Esophagus = 866, Heart = 515, Kidney Cortex = 49, Liver = 138, Lung = 327, Muscle = 474, Pancreas = 189, Prostate = 140, Skin = 1088, Small Intestine = 118, Spleen = 132, Testis = 216). (A), all commonly expressed protein coding genes as detected in uEV and GTEx tissues (n = 10,350); (B), a subset of “kidney depleted genes”, i.e. genes normally not expressed in kidney (n = 200 present in uEV+GTEx combined dataset out of 4,631, details in Methods); (C), “specific kidney enriched genes”, a subset of kidney enriched genes (n = 77, as shown in S5) that are reported to be detected in kidney and only in some other human tissues (see STAR Methods). (D) Deconvolution analysis of kidney cell types for the uEV mRNAs (T1D discovery cohort, n = 72) using adult kidney single-nucleus sequencing data as a ref. 25. The boxplots depict the interquartile range, median and minimum/maximum summary values. Counts per million (CPM); genotype-tissue expression (GTEx); principal component (PC); type 1 diabetes (T1D); urinary extracellular vesicles (uEV).

We next compared the expression profile of uEV mRNAs with the profiles of 17 other human tissues obtained from the GTEx database (https://www.gtexportal.org/home/datasets) (all male, Figures 3A–3C). To infer proximity between the profiles, we performed principal component analysis (PCA) analysis using expression levels of three sets of genes; 1) all protein coding genes expressed in both uEV and the 17 reference human tissues (n = 10,350), 2) “kidney-depleted genes” as a negative control, and 3) “specific kidney-enriched genes” (n = 77, as shown in Figure S5A). PCA analysis using gene expression data of all common gene detected in uEV showed that uEV samples clustered together with the kidney cortex tissue samples and away from other tissues suggesting close similarity of the mRNA expression patterns between uEV and kidney (Figure 3A). The PCA analysis of kidney-depleted genes showed proximity of prostate samples with uEV samples (Figure 3B). We observed a similar pattern, clustering of kidney cortex and uEV samples, when using specific kidney-enriched genes in the PCA analysis (Figure 3C). The deconvolution analysis of uEV mRNAs using adult kidney single-nucleus sequencing reference data^25^ showed that we could infer cellular fraction for all major kidney cell types with highest fraction for proximal tubular cells (Figure 3D) using cell type marker data as described in Sc-Type database.^27^

In summary, we detected expression of a high number of kidney-enriched genes in uEV and generally a lack of expression of genes not known to be expressed in kidney. The overall expression pattern of protein coding genes in uEV also showed close similarity with kidney and particularly proximal tubule, suggesting that they contribute majority of mRNAs detected in uEV.

### Genome-wide uEV mRNA analysis revealed novel candidate markers for T1D DKD in discovery phase

The distribution of gene expression of all the genes appeared similar for both discovery T1D cohorts despite the differences in urine collection protocols (Figure S5B). To further test for differences in uEV mRNA profiles, we performed PCA of all (n = 10,596) robustly expressed genes. We observed no systematic differences as 67 out of 72 samples clustered together (Figure S5C). However, the five outliers were removed from downstream case-control analysis.

**Figure 4:**
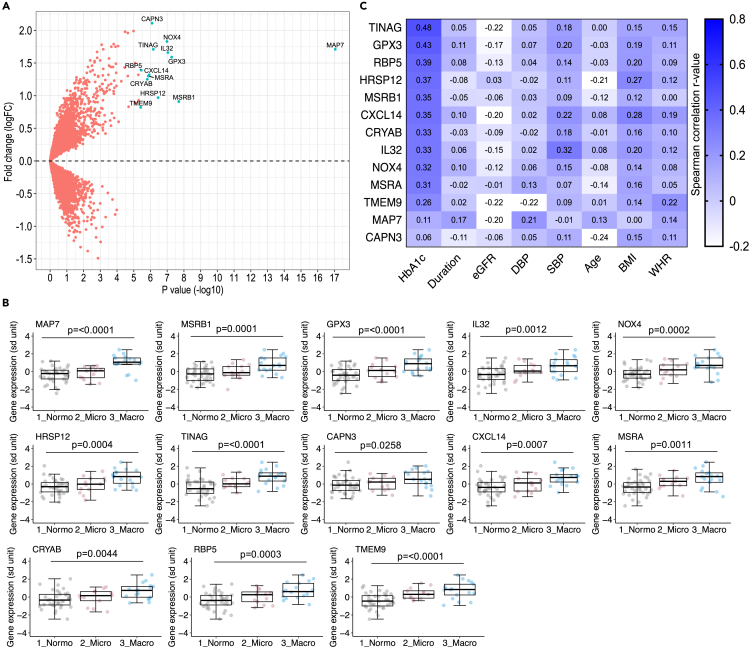
Upregulation of 13 uEV transcripts in albuminuria and their correlation with clinical parameters in T1D discovery cohort (A) Global differential expression analysis consisting of 10,596 genes and comparing macro- (n = 17) and normoalbuminuric T1D (n = 37) groups of the discovery cohort. Plot depicts the names of 13 differentially expressed genes that remained significant after multiple testing (p ≤ 3.85x10^−6^, Bonferroni threshold). (B) The boxplots illustrate the mRNA expression level distribution (sd unit) of 13 differentially expressed genes in the macro- and normoalbuminuric T1D groups (as in A) along with the microalbuminuric T1D group (n = 13). (C) Heatmap depicting the strength of correlation between expression level of the 13 genes in uEV with eight continuous clinical parameters in the T1D individuals (n = 65–67 samples, including 35–37 with normo-, 13 with micro- and 17 with macroalbuminuria). The analysis (Normo vs. Macro) in A was performed based on count data using generalized linear models adjusting for age, body-mass index, diabetes duration and urine collection protocols (overnight and 24 h). In B, the comparisons were performed using one-way ANOVA. The gene expression values (log2CPM) were inverse normally transformed and converted to *Z* score unit (sd unit). The boxplots depict the interquartile range, median and minimum/maximum summary values. In C, all data were transformed using inverse rank transformation before Spearman’s correlation analysis (two tailed). Fold change (FC); log2 normalized counts per million (log2CPM); macroalbuminuria (Macro); microalbuminuria (Micro); normoalbuminuria (Normo); type 1 diabetes (T1D); urinary extracellular vesicles (uEV).

We performed hypothesis-free global differential gene expression analysis using mRNA expression profiles of uEV (n = 10,596 genes) and comparing macroalbuminuric and normoalbuminuric T1D individuals (n = 17 and n = 37, respectively, mRNAseq PCA quality passed) (Table S2 and Figure 4A). The analysis revealed differential expression of 13 genes (*MAP7*, *MSRB1*, *GPX3*, *IL32*, *NOX4*, *HRSP12*, *TINAG*, *CAPN3*, *CXCL14*, *MSRA*, *CRYAB*, *RBP5,* and *TMEM9*) with robust significance (p ≤ 3.85 × 10^−6^) after accounting for multiple correction of all genes (Figure 4A). Interestingly, the expression level of all the differentially expressed genes was upregulated in the uEV of macroalbuminuric T1D individuals (Figure 4B).

Having shown a difference between normo- and macroalbuminuric individuals, we then studied the expression of the identified 13 genes also in the microalbuminuric group (n = 13, mRNAseq PCA quality passed). The gene expression tended to increase from normo-to micro-to macroalbuminuria (Figure 4B). While the difference between normo- and microalbuminuric individuals was not statistically significant for all the genes, the combined micro- and macroalbuminuria group differed significantly from the normoalbuminuric group (Table S6).

We also reanalyzed the uEV mRNA profile of the top 13 candidate genes by stratifying the same T1D individuals based on CKD (chronic kidney disease) stages, irrespective of albuminuria, using retrospective eGFR data (>5 years) from routine laboratory follow-up testing (Table S4). This confirmed significant (p < 0.004) upregulation of all 13 differentially expressed genes also in T1D individuals with CKD stage ≥3 compared with CKD stage ≤2 (Table S6).

To technically validate the differential expression results from NGS, we studied the expression of five of the top differentially expressed genes (*MAP7*, *GPX3*, *IL32*, *NOX4*, *HRSP12*) using Taqman quantitative PCR (qPCR) assays in the same set of samples. The qPCR- and mRNAseq-based expression values were mostly highly correlated (Figure S6). Similar to the mRNAseq results, the qPCR-based expression levels were significantly (p ≤ 0.005) upregulated in macroalbuminuric T1D individuals and somewhat less upregulated in microalbuminuric subjects compared to those with normoalbuminuria (Figure S6).

### Differentially expressed uEV mRNAs correlated with hyperglycemia and showed co-expression pattern similar to kidney samples

We studied whether the expression level of the 13 differentially expressed genes, considered as candidate marker genes for T1D DKD, correlated with clinical parameters contributing to DKD progression (hyperglycemia and hypertension) at or close to the time of urine collection (Figure 4C).

**Figure 5:**
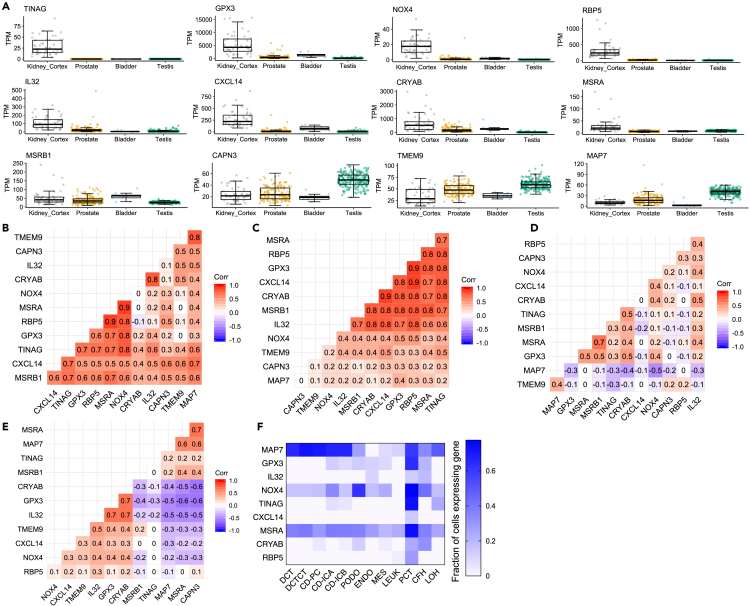
Resemblance in gene expression signature of the top DKD candidate genes between uEV and human kidney tissue (A) Expression levels (interquartile range, median and minimum/maximum summary values) of the genes-shown to be differentially expressed in uEV from macro-vs. normoalbuminuric patients (12 out of 13 genes depicted)- in male urogenital organs obtained from the GTEx reference database (tissues from men only; kidney-cortex, n = 49; prostate, n = 140; bladder, n = 9; testis, n = 216). (B–E), Pairwise gene expression correlation (Spearman’s correlation analysis) among the differentially expressed genes (n = 12) in male urogenital organs (from GTEx as in A) or uEV (n = 72, both T1D cohorts): B- kidney-cortex; C- uEV; D-prostate; E-testis. (F) Heatmap showing the fraction of specified kidney cells expressing the nine differentially expressed genes. Data is from single cell sequencing of human diabetic kidney and obtained from Wilson et al.^25^ The assessed kidney cell types were: PCT, proximal convoluted tubule; CFH, complement factor H; LOH, loop of Henle; DCT, distal convoluted tubule; CT, connecting tubule; CD, collecting duct; PC, principal cell; IC, intercalated cell; PODO, podocyte; ENDO, endothelium; MES, mesangial cell; LEUK, leukocyte. Correlation (Corr.); diabetic kidney disease (DKD); genotype-Tissue Expression (GTEx); transcripts per million (TPM); urinary extracellular vesicles (uEV).

Most of the differentially expressed candidate genes (11 out of 13), except *MAP7* and *CAPN3*, showed a positive correlation with HbA_1c_ (p < 0.05, Figure 4C). Of note, the strongest correlation (p ≤ 0.001) was observed for the candidate genes known to be highly expressed in kidney (*TINAG*, *GPX3,* and *RBP5*) compared to other human tissues (Figure 5A). In a subanalysis that included samples from normoalbuminuric individuals only (n = 37), we also saw a trend of positive correlation between expression levels of 10 candidate genes and HbA_1c_ suggesting that the observed correlations in the whole T1D cohort were not only driven by cases with clinically confirmed DKD (Figure S5D).

We next explored the origins of the differential uEV transcripts by inspecting their co-expression patterns in uEV and GTEx-an external human tissue expression reference database (https://www.gtexportal.org/home/datasets). Considering the urogenital organs previously established to be important contributors of uEV (Figure 3), the expression level of 8 out of 12 candidate marker genes (present in GTEx dataset) were higher in kidney cortex compared to prostate, bladder, and testis samples in the GTEx datasets (male only, Figure 5A). By pairwise correlation analysis, the co-expression pattern of the candidate genes in uEV resembled the pattern in kidney cortex when focusing on most of the kidney-enriched genes (*MSRA*, *RBP5*, *GPX3*, *CXCL14*, *TINAG*, *NOX4*, *IL32,* and *CRYAB*), but also in case of many of the non-kidney enriched candidate genes (*MSRB1*, *CAPN3*, *TMEM9,* and *MAP7)* (Figures 5B–5E). Further, we utilized single nuclei RNAseq data (>23,000 nuclei) from human diabetic kidney tissue,^28^ to look up the expression levels of the candidate genes in different cell types of the kidney. Most of the candidate genes (6 out of 9 genes with cell type specific expression data available showed highest cellular percentage for the proximal convoluted tubule (PCT) cells expressing these genes (Figure 5F).

In summary, expression analysis of candidate marker genes for T1D DKD suggested that hyperglycemia may contribute to their higher expression in uEV samples. The main contributors of the candidate marker genes to uEV could be the kidney PCT cells.

### uEV mRNA-based stress score and its association with DKD in multiple cohorts

**Figure 6:**
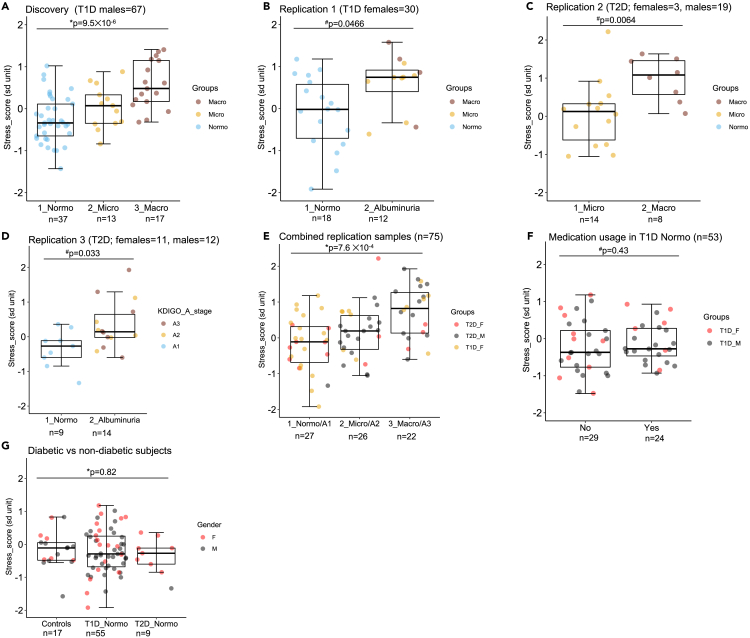
Transcriptomic stress score is associated with albuminuria status in different diabetic cohorts (A) Expression level of the transcriptomic stress score in different albuminuria groups of the T1D discovery cohort. Stress score was constructed based on six differentially expressed uEV mRNAs, among top 13 candidate genes, involved in cellular stress responses (genes *GPX3*, *NOX4*, *MSRB1*, *MSRA*, *HRSP12,* and *CRYAB*). (B–E) Stress score in different albuminuria groups from three different diabetic replication cohorts, B- Replication 1 (T1D female cohort; FinnDiane study), C- Replication 2 (T2D cohort; DIREVA study) and D- Replication 3 (T2D cohort; iBEAt study), and a combined analysis of all three replication cohorts in E. (F) Stress score in normoalbuminuric individuals from all discovery and replication cohorts divided based on medication usage. (G) Stress core in non-diabetic (age; mean ± sem = 43.67 ± 4.86) and diabetic (T1D and T2D) subjects with normoalbuminuria (Table S3). The boxplots depict the interquartile range, median and minimum/maximum summary values. n = numbers as depicted in figure panels; # Mann-Whitney u test; ∗ Kruskal-Wallis test.

All or most of the 13 differentially expressed candidate marker genes for T1D DKD showed common characteristics: 1) upregulation in DKD individuals (Figures 4B), 2) positive correlation with hyperglycemia (Figure 4C), and 3) higher expression level in kidney cortex and proximal tubular cells (Figure 5). This suggested that also the underlying disease mechanism responsible for these common characteristics might be common. A literature search indicated that six of the main 13 DKD candidate genes executed functions in cellular stress responses, namely 1) oxidative stress related responses (*GPX3*, *NOX4*, *MSRB1*, *MSRA*) and 2) protection of cellular proteins during stress (*HRSP12* and *CRYAB)* (Table S7). In addition, the expression level of these six genes showed a positive correlation with HbA_1c_ (Figure 4C) and co-expression with each other (Figure 5C). Hyperglycemia-induced stress in kidney cells including the PCT is considered as an important disease mechanism contributing to DKD progression.^29^ Thus, we speculated that these six genes may be involved in hyperglycemia-induced stress responses of the kidney and that their expression level in uEV could reflect change in kidney function of diabetic individuals. To test this hypothesis, we derived a transcriptional stress score based on average normalized expression level of those six genes in uEV (*GPX3*, *NOX4*, *MSRB1*, *MSRA*, *HRSP12,* and *CRYAB*, see STAR Methods for statistics) to test association with DKD (defined by albuminuria). These six genes belong to group of top 13 transcriptome-wide significant DKD candidate genes as shown in Figure 4A. As expected, the stress score had a clear increasing trend from normo-to macroalbuminuria (Figure 6A) and showed a positive correlation with HbA_1c_ (r = 0.41) measured at the time of urine sample collection in T1D discovery study but not with eGFR, systolic and diastolic blood pressure (SBP and DBP) measured at the same time (Figure S7A). In support to the pathophysiological roles of the stress score genes, pathway enrichment (gene ontology) analysis of all nominally significant differentially expressed genes between T1D macro- and normoalbuminuria groups also suggested changes in oxidation-reduction as well as transporter and EV-linked activities (p < 0.05, logFC>1, n = 130 genes, Table S8).

We attempted to replicate the association of the stress score with albuminuria status—as seen in discovery phase—in three independent replication cohorts including one T1D (women only) and two T2D cohorts (men and women) (Figures 6B–6E, Table S3). All three replication cohorts showed higher expression level of the stress score in diabetic individuals with albuminuria compared to normoalbuminuria (p = 0.046 to 0.0068) (Figures 6B–6D). Combining all three replication cohorts yielded a significant difference between groups (Figure 6E, n = 75, p = 7.6 ✕10^−4^) following a similar pattern as in discovery phase (Figure 6A, n = 67, p = 9.5 ✕10^−6^). Using discovery and validation cohorts, we did not see any difference in the stress score levels between 1) T1D normoalbuminuria groups treated with ACE (angiotensin-converting enzyme) inhibitors and/or Beta blockers vs. not treated (Figure 6F) nor between 2) T1D and T2D normoalbuminuria groups vs. non-diabetic controls (Figure 6G).

### uEV mRNA-based stress score as early marker for long-term eGFR decline

**Figure 7:**
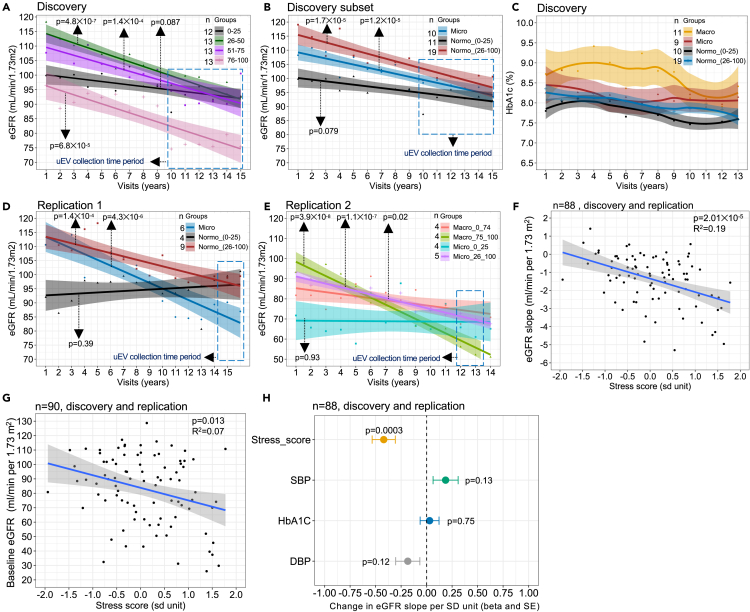
Stress score as an early marker for longitudinal decline of kidney function in diabetic individuals (A) The T1D discovery cohort was stratified into quartiles based on uEV stress score. Change of eGFR over 15 sequential visits (mean/year and slope) was analyzed retrospectively from the time of uEV collection (data available for n = 51). (B) Similar analysis in normoalbuminuric individuals of T1D discovery cohort stratified into lowest stress quartile (≤25%) or higher quartiles (>25%). Microalbuminuric group without any stress score stratification is shown as a positive control group of early DKD. (C) Longitudinal HbA1c change in normoalbuminuric individuals of T1D discovery cohort stratified into lowest stress quartile (≤25%) or higher quartiles (>25%). Clinically confirmed micro- and macroalbuminuria groups are shown as positive controls of early and late DKD, respectively. (D) Longitudinal eGFR change in replication cohort 1 (T1D female cohort; FinnDiane study) stratified as in B. (E) Longitudinal eGFR change in replication cohort 2 of DKD patients (T2D cohort; DIREVA study) stratified into the lowest and the respective highest stress score quartile(s) and shown separately for macro- and microalbuminuria groups. (F and G) Association of stress score with F- eGFR slope and G-baseline eGFR (measured at the time of uEV collection) in all samples. (H) association of stress score (sd unit) and other know causal clinical risk markers (in sd unit) with eGFR decline (sd unit change per year) independent of each other and baseline eGFR in all samples. Linear regression analysis after inverse normal transformation. In H, model consisted of stress score, DBP, HbA1C, SBP and eGFR at time of uEV collection. n = numbers as depicted in figure panels. DBP; diastolic blood pressure, eGFR; estimated glomerular filtration rate, HbA1C; hemoglobin A1C, SBP; systolic blood pressure.

Next, we tested whether the uEV stress score measured at one time point could reflect a longitudinal change in kidney functions. We stratified the T1D discovery cohort into quartiles (0–25, 25–50, 50–75, 75–100) based on the stress score and compared annual change in clinical parameters between them over time. To compare the annual eGFR change, we collected long-term eGFR data retrospectively (15 visits, from 2004 to 2018) for most of the diabetic individuals (available for 51 of 67) from clinical laboratory databases. The lowest stress score quartile (0–25) showed a minimal decline in the eGFR per year (slope_(mL/min/1.73 m2)_±SE; −0.58 ± 0.19) (Figure 7A). In contrast, the annual eGFR decline was >2-fold steeper in the highest quartile (75–100; −1.55 ± 0.27). Interestingly, the 25–50 percentile group—the group with a moderate increase in stress core— showed an even faster decline (−1.61 ± 0.17), which was derived from a higher eGFR at the retrospective starting points (visits 1–3) but no comparative difference in the recent years (visits 10–15). This suggests the mid stress group may have undergone hyperfiltration unlike the low stress group.

As the stress score was defined based on the change in the macroalbuminuria group, we performed a subanalysis of the long-term decline in eGFR excluding them. Hence, among the normoalbuminuric T1D individuals, we compared those within the lowest stress score quartile with those within the three higher quartiles (0–25 and >25) as well as with all microalbuminuric T1D individuals (regardless of the stress score) as a positive control, i.e., the group having early clinically confirmed T1D DKD. The normoalbuminuric group with the lowest stress score (0–25) showed only a minimal annual decline (−0.59 ± 0.19) in the eGFR compared to those with mid-to-high stress score (>25; −1.34 ± 0.20), which did not markedly differ from the microalbuminuria group (−1.08 ± 0.16 (Figure 7B). Similar analysis of longitudinal HbA_1c_% (13 years) did not show notable differences between the normoalbuminuria groups having low or mid-to-high stress score (0–25 and >25) (Figure 7C), but the most recent HbA_1c_ level measured at the time of uEV collection was higher for the mid-to-high (>25) stress score group similar to the microalbuminuria group (Figure S7B). In T1D women’s replication cohort, the eGFR again declined fast (−1.16 ± 0.22) in the mid-to-high stress score (>25) group as in the microalbuminuria group (−2.03 ± 0.28), whereas we observed no significant eGFR decline (0.26 ± 0.29) in the normoalbuminuric low stress group (0–25) (Figure 7D). Further, an ROC (receiver operating characteristic) analysis for predicting at least ≤ -0.5 annual eGFR decline in the normoalbuminuric individuals (n = 13) from the same T1D replication cohort revealed that the stress score was more informative (AUC = 0.83) compared to other clinical parameters (Figure S7C).

We also looked how stress score correlated with the eGFR decline in groups with clinically established DKD. The long-term eGFR data was available for one T2D DKD replication cohort (n = 17 out of 22). When separating the T2D cohort into macro- and microalbuminuria groups, the respective lowest stress groups with sufficient patient numbers for analysis showed a minimal decline (T2D-micro, 0–25, −0.05 ± 0.57; T2D-macro, 26–74, −0.98 ± 0.37) compared to their respective higher stress groups (T2D-micro, 26–100, −1.80 ± 0.16; T2D-macro, 75–100, −3.55 ± 0.29) (Figure 7E). Further, an ROC analysis for predicting fastest decliners (≤-3.07) in all DKD replication cohorts (for available T1D and T2D) revealed that the stress score was most informative (AUC = 0.79) compared to other clinical parameters (Figure S7D).

Overall, in the combined analysis-of discovery and replication cohorts regardless of diabetes type and albuminuria status—the stress score was associated (p = 2.01✕10^−5^) with eGFR decline rather than eGFR at the time of uEV collection, which remained significant (beta = −0.37 ± 0.09 sd unit, p = 0.0003) after adjusting for other known clinical parameters (Figures 7F–7H).

The study showed that a lower uEV stress score, i.e., a lower level of the candidate mRNAs in uEV samples, associates with a minimal long-term decline of kidney function. Conversely, a higher secretion of stress markers via uEV and thus a higher stress score, associates with a faster decline of kidney function in diabetic individuals. This suggests that these genes could be putative candidate DKD marker genes involved in the response of the kidney to the stress caused by hyperglycemic conditions.

## Discussion

Extracellular RNAs originating from the kidney and passed to urine inside EV could serve as a non-invasive proxy for a biopsy, a liquid kidney biopsy. Overall, we detected expression of >10,000 genes despite focusing on the most commonly expressed genes (>90% samples). They define the most stable EV mRNA transcriptome in population scale covering particularly male T1D population. The expression of large gene numbers in uEV is in line with results from some recent smaller sequencing studies.^30^^,^^31^^,^^32^^,^^33^ Interestingly, irrespective of the degree of albuminuria, the uEV showed only a small subset of transcripts (<5%) not known to be expressed in the kidney compared to a high proportion (>50%) of kidney-enriched genes and a genome-wide expression pattern similar to kidney tissue. This suggests that kidney is the main contributor of uEV and that the glomerular filtration barrier limits the access of EV from non-urinary tract organs into the urine even during DKD. Nevertheless, as we did detect some non-kidney expressed genes, we cannot totally exclude the possibility of a low degree EV transfer from circulation.^34^ Our results also agree with previous data showing uEV mRNAs from other urogenital organs including prostate.^17^

EV field is still in its infancy with non-standardized workflows for urine, particularly when considering EV mRNAs compared to miRNAs in DKD.^35^ Thus, we needed to test several pre-analytical and analytical factors. Specifically, we assessed the impact of the urine collection methods used in the clinic, 24-h or ON, which can affect the uEV concentration or cargo and thereby the detection sensitivity. We confirmed that ON urine, which is easier to collect, produced an almost similar mRNA profile and expression level of kidney enriched genes as the 24-h urine. Additionally, our whole uEV pipeline was reproducible, as it gave similar mRNA transcriptomes from the technical replicates processed at 1–5 months intervals through the pipeline. This suggests that urine storage at −80°C preserves uEV mRNAs well. Further, the degree of variation due to technical steps that are often assumed to cause problems, such as uEV isolation by ultracentrifugation^36^ or sequencing in different batches,^37^ was not significant here. We also validated the mRNAseq results with qPCR for the top differential genes.

Despite our focus on men’s samples in an attempt to reduce heterogeneity in the discovery phase, we also explored the differences in the uEV RNA between men and women. In uEV RNA samples from women, we observed more frequently signs of RNA degradation and DNA contamination than in samples from men. In agreement with our results, it has been reported that DNA and RNA quantities are higher in uEV samples derived from women compared to men.^38^^,^^39^ Further, while urinary EV DNA appears to be mainly located outside of the EV,^40^ it is known that RNA outside of the EV is prone to degradation.^41^ Thus, a plausible explanation for our findings is that the samples from women contained more often non-vesicular degraded RNA and DNA e.g. from remnants of epithelial cells. However, we overcome this difficulty with women’s samples by introducing the new quality control criteria (Figures 1 and S1). We also confirmed that the differences in uEV transcriptomes were minimal between sexes when focusing on kidney-enriched genes and the stress score (Figures 6 and S4). This technical information is provided as a workflow (methods) and should assist in standardization efforts from biobanking activities of urine to future large-scale uEV mRNA research.

The study revealed putative marker genes that are prevalently expressed in PCT for DKD in T1D individuals. The important roles of PCT in kidney dysfunction (DKD and chronic kidney disease) is also supported by kidney tissue bulk^7^ and single cell RNA sequencing^28^^,^^42^ and genetic studies.^43^ Remarkably, now uEV provide an opportunity to assess the expression of some highly PCT-enriched genes (e.g. *TINAG*, *CXCL14, and RBP5)* (Figure 5) non-invasively in T1D individuals progressing toward DKD.

Based on the known functions of the DKD candidate marker genes, it appears that many associate with kidney diseases, and our stress score genes, particularly *GPX3*, *HRSP12*, *MSRA*, *MSRB1,* and *CRYAB*, protect the cell and/or cellular proteins during stress (Table S7). They thus may have a reno-protective role by mitigating hyperglycemia-induced stress in the kidney. *GPX3* is one such antioxidant enzyme mainly produced in PCT and involved in relieving oxidative stress by neutralizing hydrogen peroxide.^44^ The *GPX3* and *MSRB1* belong to the selenoprotein class.^45^ Selenium deficiency induces oxidative stress in murine kidney cells.^46^ Recently, enhancing the *GPX3* activity by selenium supplementation was shown to rescue kidney function in T2D human subjects.^47^ Gene *NOX4*, also one of the stress score genes, has many roles in DKD ranging from detrimental to protective and its protein level was recently shown to be decreased in PCT during CKD.^48^^,^^49^ Increased EV secretion during various types of stress, including oxidative stress (mechanical stretch, hypoxia, hyperglycemia, inflammation, acidosis) have been reported and thus could contribute to the “leaking” of these reno-protective kidney transcripts with putative roles in managing hyperglycemic stress in diabetic kidney.^50^^,^^51^^,^^52^ One mechanistic explanation for increased EV secretion could be that the impaired intracellular EV degradation via defective autophagy in DKD would lead to increased EV secretion,^48^^,^^53^ as the two systems appear closely linked, which is supported by accumulating data from cell culture studies.^54^^,^^55^^,^^56^ Alternatively, or additionally, the increased transcript levels in uEV could be caused by lack of uptake and disturbed intra-renal signaling.^13^^,^^57^ Overall, further studies would help to confirm whether increased transcript secretion via uEV in diabetic individuals causes a decrease in the level of reno-protective proteins in the kidney or whether the secretion is just a consequence of an ongoing compensatory mechanism in a hyperglycemic kidney.

Clinically, the most interesting finding in our study was that the uEV-based stress score correlated with long-term decline of kidney function (eGFR) in diabetic individuals even before manifestation of albuminuria (Figures 7B–7D). The stress score thus has potential for non-invasive early identification of individuals with declining kidney function and a greater risk of developing DKD. Indeed, our study suggests uEV may be sensitive indicators of the DKD development due to the coupling of their biogenesis to stress - hyperglycemic stress causing oxidative cellular stress - and possibility for tracking their rich transcriptomic information to the cell-of-origin, notably to the PCT.

### Limitations of the study

The main limitations of our study were 1) low sample size of individual groups, 2) focusing on male subjects in the discovery phase (due to expected higher heterogeneity in women, which could have had an impact considering the low sample size). However, given the novelty of the methods—whole transcriptome EV mRNAseq—this is the largest study until now with inclusion of two different T1D cohorts, two T2D cohorts, healthy controls, and detailed long-term retrospective clinical data. Additionally, we define a robust uEV transcriptome of >10,000 genes, show the position of uEV on tissue map, and provide evidence that uEV pipelines can be reproducible and applicable for high-impact large-scale biomarker studies. Our data resource should further help researchers in studying the mRNA landscape and kidney function in clinical uEV samples.

In summary, the study showed the utility of uEV transcriptomics in capturing the gene expression patterns of the kidney and discovered T1D DKD candidate marker genes including putative reno-protective genes. Because these genes showed initial promise for T2D DKD as well, they appear suitable for development as broadly applicable novel biomarkers or therapeutic targets for DKD. Thus, we conclude that uEV offer a promising liquid kidney biopsy of DKD.

## Consortia

On behalf of iBEAt study investigators- Kim M Gooding, Chrysta Lienczewski, Niina Koivuviita, Anna-Maria Dutius Andersson, Kanishka Sharma, Paola Pontrelli, Virva Saunavaara, Loreto Gesualdo, Nicolas Grenier, Angela C Shore, Maria F. Gomez, Steven Sourbron, Francesca Conserva, Andrew Forbes Brown, Kelly Wroe, Corrine Castermans, Sophie Regueme, Iris Friedli, Annalisa Perna, Kywe Kywe Soe, Joao Periquito, Dinesh Selvarajah, Carmen Hurtado Del Pozo, Johanna Paivarinta, Anil Karihaloo, Mark Gilchrist, Marlena Maziarz, Norberto Perico, Lars Johansson, Kunihiko Aizawa, Cherry Coupland, Paul Hockings, Rosamonde E Banks, Kelly Wroe.

## STAR★Methods

### Key resources table

**Table:** 

REAGENT or RESOURCE	SOURCE	IDENTIFIER
**Antibodies**		

Mouse monoclonal against CD9	Santa Cruz	Cat# SC-13118; RRID: AB_627213
Mouse monoclonal against Podocalyxin (clone 3D3)	Novus Biologicals	Cat# NBP2-25219; RRID: N/A

**Biological samples**		

Urine, non-diabetic and type 1 diabetic individuals	Finndiane study	http://www.finndiane.fi/
Urine, Type 1 and 2 diabetic individuals	DIREVA study	https://www.helsinki.fi/en/researchgroups/genetic-and-metabolic-characterization-of-diabetes/direva-and-beat-dkd
Urine, Type 2 diabetic individuals	iBEA tstudy	Clinicaltrials.gov -NCT03716401, Gooding et al.^58^
Urine, non diabetic individuals	Botnia study	https://www.helsinki.fi/en/researchgroups/genetic-and-metabolic-characterization-of-diabetes/research-and-projects/botnia-family-study-and-botnia-prospective-study
Urine, non-diabetic individuals, n= 6	HUB study	Puhka et al.^17^

**Chemicals, peptides, and recombinant proteins**		

Protease Inhibitor Cocktail Set III	Calbiochem	Cat# 539134
Complete ULTRA, Mini, EDTA-free, Roche	Roche	Cat# 11 836 170 001

**Critical commercial assays**		

miRNeasy micro	Qiagen	Cat# 217084
miRNeasy mini	Qiagen	Cat# 217004
Agilent RNA 6000 Pico Kit	Agilent	Cat# 5067-1513
SMART-Seq® v4 Ultra™ Low Input RNA Kit for Sequencing	Takara BIo Inc.	Cat# 634889
Nextera XT DNA Library Preparation Kit (24 samples)	Illumina Inc.	Cat# FC-131-1024
Nextera XT Index Kit (24 indexes, 96 samples)	Illumina Inc.	Cat# FC-131-1001
NextSeq 500 v2 High Output PE 150 Cycle	Illumina Inc.	Cat# FC-404-2002
LabChip GX Touch HT High Sensitivity assay	PerkinElmer	Cat# CLS760672
High Sensitivity D5000 ScreenTape system	Agilent	Cat# 5067-5592
SMART-Seq® stranded kit	Takara BIo Inc.	Cat# 634444
High Sensitivity DNA Kit	Agilent technologies	Cat# 5067-4626
Qubit™ 1X dsDNA HS Assay Kit	Thermo Fisher Scientific, Inc.	Cat# Q33230

**Deposited data**		

Raw gene count data of both T1D cohorts	This paper	Table S9
Raw gene count data of technical comparison samples	This paper	Table S10
Web resource for uEV mRNA data in T1D discovery cohorts	This paper	https://uev-mrna.rahtiapp.fi
The reference human genome annotation	GENCODE	https://www.gencodegenes.org/human/#
GTEx Analysis V8 (dbGaP Accession phs000424.v8.p2)	GTEx	https://www.gtexportal.org/home/
Kidney enriched gene list	The Human Protein Atlas (Version 20)	https://v20.proteinatlas.org./humanproteome/tissue/kidney

**Oligonucleotides**		

MAP7 (Hs01010631_m1)	Applied Biosystems	Cat# 4448892
MAP7 (Hs01009609_m1)	Applied Biosystems	Cat# 4448892
GPX3 (Hs00173566_m1)	Applied Biosystems	Cat# 4453320
GPX3 (Hs01078668_m1)	Applied Biosystems	Cat# 4453320
IL32 (Hs00170403_m1)	Applied Biosystems	Cat# 4453320
IL32 (Hs00992441_m1)	Applied Biosystems	Cat# 4453320
NOX4 (Hs04980924_m1)	Applied Biosystems	Cat# 4453320
NOX4 (Hs01379108_m1)	Applied Biosystems	Cat# 4453320
HRSP12 (Hs01066170_m1)	Applied Biosystems	Cat# 4448892
GAPDH (Hs99999905_m1)	Applied Biosystems	Cat# 4453320

**Software and algorithms**		

STAR (STAR-2.6.1d)	Dobin et al.^59^	https://github.com/alexdobin/STAR
featureCounts (Version 1.6.4)	Liao et al.^60^	https://subread.sourceforge.net/
edgeR	Robinson et al.^61^	https://bioconductor.org/packages/release/bioc/html/edgeR.html
RSeQC	https://rseqc.sourceforge.net/	https://rseqc.sourceforge.net/
Database for Annotation, Visualization and Integrated Discovery (DAVID) v6.8	Huang et al.^62^	https://david.ncifcrf.gov/
BisqueRNA	Jew et al.^63^	https://cran.r-project.org/web/packages/BisqueRNA/index.html
ScType	Ianevski et al.^27^	http://session.asuscomm.com/

### Resource availability

#### Lead contact

Further information and requests for resources and reagents should be directed to and will be fulfilled by the Lead Contact, Om Prakash Dwivedi (om.dwivedi@helsinki.fi).

#### Materials availability

This study did not generate new unique reagents.

### Experimental model and subject details

#### Human subjects and clinical protocol

Urine samples and clinical data were collected in the 1) nation-wide prospective FinnDiane (Finnish Diabetic Nephropathy) Study targeting risk factors for DKD in type 1 diabetes (T1D), and 2) the DIREVA study (Diabetes Registry in Vaasa), inviting all diabetic individuals living in the Vaasa Central Hospital District to participate since 2007 and 3) iBEAt study (Prognostic imaging biomarkers for diabetic kidney disease, Gooding et al.^58^), 4) Botnia study (Dwivedi et al.^64^) and 5) HUB study (Helsinki Urological Biobank and the SalWe “Get It Done” research projects, Puhka et al.^17^). The studies followed the principles of the Declaration of Helsinki and all participants gave an informed consent prior to participation. The study protocol for this substudy of the FinnDiane and DIREVA studies was approved by the Ethics Committee of the Turku University Hospital. The study protocol of the FinnDiane Study has also been approved by the Ethical Committee of the Helsinki and Uusimaa Hospital District (FinnDiane 163/E5/04, 199/E5/05, 491/E5/2006; 238/13/03/00/15), and that of DIREVA, by the Ethical Committees of the Vaasa Central Hospital (6/2007) and Turku University Hospital (48/1801/2014; 116/1805/2016). Descriptions including ethics approvals for iBEAt, Botnia and HUB studies are available in the above referred publications.

In the FinnDiane Study, type 1 diabetic men aged 18-70 years were recruited. T1D was defined as an onset of diabetes before the age of 40 and insulin treatment initiated within the first year after diagnosis. In spurious cases, C-peptide levels (<0.2 mmol/l) were required and medical files were reviewed. In DIREVA, male individuals with clinical T1D (aged 30-60 years) or type 2 diabetes (T2D) (aged 56-82 years) diabetes with different degrees of albuminuria and no history of pancreatitis were invited. Information on diabetes heredity, history of pancreatitis or gestational diabetes was also collected to verify the diagnosis of T1D. Study subjects were investigated at the local study centers, where trained research nurses or physicians collected information using standardized questionnaires on, among others, sex, age, age at onset of diabetes, duration of diabetes, micro- and macrovascular complication profile and current medication. The nurses measured height, weight, waist and hip circumference in light clothing, and body-mass index (BMI) and waist- to-hip ratio (WHR) was calculated. The blood pressure was measured twice with a 5 min interval from the right arm of a sitting person after an initial rest. The mean of the two measurements was calculated and used in the analyses. Venous blood samples were taken for the assessment of HbA_1c_, blood lipids and lipoproteins (total and HDL-cholesterol and triglycerides), creatinine, fS-C-peptide, fP-Glucose, S-GAD-antibodies (DIREVA) and DNA. The samples were taken either fasting or a minimum of two hours after a light meal. In addition, retrograde data on HbA_1c_, serum and plasma creatinine for estimation of glomerular filtration rate (eGFR using the CKD-EPI formula) (Levey et al.^65^), urine albumin excretion rate (AER) and urine albumin-creatinine ratio (ACR) were obtained from hospital laboratory databases for the validation of the albuminuria status as well as for the longitudinal analyses.

Altogether, our study included 72 men with T1D (FinnDiane and DIREVA studies) in discovery phase and three replication cohorts consisting of one T1D study and two T2D studies (Table S3). In addition, we included non-diabetic individuals from Botnia (n=13) and HUB studies (n=6) for comparative analysis. The participants were stratified into normo-, micro- and macroalbuminuria groups. In the FinnDiane cohort, the stratification was done according to the AER (based on 24-h urine collection) in two out of three consecutive urine collections: macroalbuminuria with AER >300 mg/24-h, microalbuminuria with AER 30-300 mg/24-h, and normoalbuminuria with AER <30 mg/24-h (Tables S1 and S2). In the DIREVA cohort, macroalbuminuria was defined as AER (based on ON urine collection) >200 μg/min or ACR (spot urine albumin/creatinine ratio) >35 mg/mmol, and microalbuminuria as AER 20-200 μg/min or ACR >3.5 mg/mmol at the last clinical visit. Normoalbuminuria was defined as AER <20 μg/min and ACR <3.5 (mg/mmol) at all of the clinical visits (Tables S1 and S2). In the iBEAt cohort, the stratification followed the KDIGO, where severely increased or macroalbuminuria was defined as ACR >25 or 35 mg/mmol (A3), moderately increased or microalbuminuria as ACR 2.5-25 or 3.5-35 mg/mmol (A2) and normoalbuminuria as ACR <2.5 or 3.5 mg/mmol (A1) for men and women, respectively. For trajectory analysis (Figure 7), we used available retrospective data on HbA_1c_ and serum or plasma creatinine from both T1D cohorts: 1) for the last 13-15 years (available for 51 T1D individuals), and 2) for the minimum of last five years (available for 66 T1D individuals). For the T2D DIREVA cohort, 14 years of retrospective data on plasma creatinine levels were available. For eGFR slope calculations using 15 years data (minimum of >8 years visit data), we used the average of all eGFR values per year for each individual. The classification into CKD stages (Table S4) was based on retrospective data of at least > 5 years eGFR measurements for each T1D individual (n=66), where at least two annual average values falling into the lowest eGFR range determined the CKD stage; stage 1-2 (eGFR >60), or stage 3-5 (eGFR <60). For the technical comparisons of uEV mRNAseq (Figures 2, S3, and S4), we also included age and sex matched non-diabetic controls (n=4) and women with T1D (for Figure S4) and normo- or microalbuminuria (n=3) from the FinnDiane Study (Table S5).

### Method details

#### Urine collection and isolation of uEV

For the discovery cohorts, the male participants collected whole voids of either 24-h (FinnDiane) or ON (DIREVA) urine into containers with Protease Inhibitor (PI) Cocktail Set III (Calbiochem, final dilution 1/1000-1/2000). The urine samples were kept in temperature ranging from ambient to +4°C during collection and delivery (the same or next day) to laboratory, where they were processed with (DIREVA) or without (FinnDiane) centrifugation at 1800g for 10 min at +4°C and frozen at -80°C. In addition, for technical comparisons, we collected similarly paired 24-h and ON urines from the same day and individuals (without centrifugation), and 24-h urine samples from which aliquots were processed in parallel with and without centrifugation (1800g, 10 min +4°C) prior freezing (FinnDiane). EV were isolated from 30 ml of urine by differential centrifugation. The urine samples were vortexed for 90s, centrifuged at 8000g for 15 min at +4°C and the resulting supernatant filtered (1.2 μm cellulose acetate, Whatman, GE Healthcare, Buckinghamshire, UK) to remove cell debris. EV were pelleted by ultracentrifugation with a SW-28 rotor at 27500 rpm (136 367g_max_, k-factor 256, Beckmann-Coulter, Inc., Brea, CA, USA) for 1h 30 min at +4°C, washed with PBS and the ultracentrifugation step repeated. EV pellets were suspended in PBS and stored in protein or DNA Low-bind tubes (Eppendorf, Hamburg, Germany) at -80°C. Further details for the isolation of uEV used in the study has been reported previously.^22^^,^^66^ For technical studies, samples from FinnDiane and DIREVA were collected and processed before freezing and for uEV isolation as above. Samples from Botnia were collected and processed similarly to DIREVA samples (i.e. overnight and with PI) and those of HUB as described (Puhka et al.,^17^ i.e. spot mid-stream urines without PI).

For the validation cohorts, participants collected either whole voids of 24-h urine (FinnDiane, T1D women), ON urine (DIREVA, T2D men and women) or first void morning urine (iBEAt, T2D men and women). DIREVA samples were collected with PI and centrifuged at 1800g for 10 min at +4°C before freezing as above, and FinnDiane samples were collected either with or completely without these steps. iBEAt samples all contained PI (Complete ULTRA, Mini, EDTA-free, Roche; half tablet per 50 ml urine) and were centrifuged at 3000g for 10 min at RT, after which citrate-EDTA buffer was added (1,0 M citrate, 0,1 M EDTA; 2,5 ml per 45 ml urine) before freezing at -80°C. The uEV were isolated from 30 ml of urine (DIREVA and part of FinnDiane) as above except using a SW-32 rotor (129 168 gmax, k-factor 276, Beckmann-Coulter) and from 8 ml of urine (iBEAt and part of FinnDiane) as described (Barreiro et al.^23^) using a 70.1 ti rotor (82 656 gmax, k-factor 202, Beckman Coulter, Inc., Brea, CA) for 2 h 30 min at 30,000 rpm at +4°C and no wash.

#### uEV protein and particle quality control

Urinary EV samples were analyzed as explained previously.^22^^,^^66^ Briefly, Western blotting of uEV marker proteins was performed using antibodies against CD9 (SC-13118, Santa Cruz) and podocalyxin (clone 3D3, Novus Biologicals). EV from equal volumes of urine (1.7 ml) and 20 μg of protein from LNCaP cell lysate control were loaded to gels. For detection, x-ray film (Ultra Cruz™ Autoradiography Film, Santa Cruz Biotechnology (SC), Dallas, TX, USA) was used.

Electron microscopy (EM) samples were prepared with a negative staining protocol. The samples were viewed with Jeol JEM-1400 (Jeol Ltd., Tokyo, Japan) at 80 kV and imaged with Gatan Orius SC 1000B CCD-camera (Gatan Inc., USA) with 4008 × 2672 px image size and no binning.

NTA was done with Nanosight LM14 (Malvern Instruments Ltd, Malvern, UK) with blue (404 nm, 70 mW) laser and SCMOS camera (Hamamatsu photonics K.K., Hamamatsu, Japan). Samples were diluted in filtered (0.1μm, Millex VV, Millipore) DPBS to obtain 40-100 particles per view. Five 30 s videos were recorded with camera level 13. The videos were analyzed using software version 3.0 (Malvern Instruments Ltd), detection threshold 5 and screen gain 10.

#### RNA preparation, mRNA sequencing and data analysis

Total RNAs from the uEV were extracted using miRNEasy mini or micro kit according to manufacturer's instructions without a DNAse treatment (Qiagen, Hilden, Germany). The only exception was that for the paired HUB urine (1 ml) and uEV samples used for technical comparisons, lysis was performed using Trizol LS (LifeTechnologies Corp., Carlsbad, CA, USA) due to its suitability for larger sample volumes. RNA profiles and concentrations were measured with RNA 6000 Pico Total RNA Kit run on Bioanalyzer 2100 (Agilent Technologies, Santa Clara, CA). For mRNAseq, the cDNA was prepared with SMART-Seq® v4 Ultra™ Low Input RNA Kit for Sequencing (Takara BIo Inc., Mountain View, CA) using 0.4 ng (HUB samples) or 1 ng (DIREVA and FinnDiane) of total RNA as the input. The cDNA libraries were prepared with Nextera XT DNA Library Preparation and Index Kits (Ilumina Inc., San Diego, CA, USA). The cDNAs and libraries were measured with High Sensitivity D5000 ScreenTape system of TapeStation (Agilent) or LabChip GX Touch HT High Sensitivity assay (PerkinElmer, USA). Paired end (75, 100, 150 bp) sequencing was performed on NextSeq 550, Novaseq 6000 or Hiseq 2000 (Illumina). RNA sequencing reads were aligned to hg38 using STAR (STAR-2.6.1d),^59^ genome annotations were obtained from the GENCODE (Encyclopedia of Genes and Gene Variants) v227 program,^67^ and read counting was done using featureCounts (Version 1.6.4) software package.^60^ Read counts were normalized using TMM (trimmed mean of M-values) method.^68^ For total RNAseq of iBEAt cohort samples, the cDNA libraries were prepared from 7 μl of total RNA using SMART-Seq® stranded Kit (Takara BIo Inc). Quality control and concentrations of individual libraries were assessed with Bioanalyzer 2100 Instrument and High Sensitivity DNA Kit (Agilent Technologies) and using the Qubit™ 1X dsDNA HS Assay Kit (ThermoFisher). RNA sequencing was performed on the Illumina NovaSeq 6000 platform (Illumina Inc.) as 107 bp cycle single end read.

#### Kidney enriched gene list, GTEx and deconvolution analysis

We used human protein atlas (version 10, resource table) to obtain the lists of 1) “kidney enriched genes” (n=413, detected in kidney and also many other tissues) with an elevated expression in the kidney compared to other tissue types, 2) “specific kidney enriched genes” (n=190) – a subset of “kidney enriched genes” (n=413) that are detected in kidney and only in some (more than one but less than one third of 37 tissues) other tissues and 3) “kidney depleted genes” (n=4631) not detected in kidney (https://www.proteinatlas.org/humanproteome/tissue/kidney). In the group of uEV samples used for technical comparisons, we detected 233 “kidney enriched genes” (≥1 count in all samples). In the two T1D discovery cohorts we detected 247 “kidney enriched genes” (≥1 count in all samples) and 77 “specific kidney enriched genes”. Additionally, in both T1D cohorts we detected only 200 “kidney depleted genes” (≥1 count in all samples) in uEV.

We used raw gene count (GTEx_Analysis_2017-06-05_v8_RNASeQCv1.1.9_gene_reads) and TPM values (GTEx_Analysis_2017-06-05_v8_RNASeQCv1.1.9_gene_tpm) from GTEx databases (GTEx analysis V8) for analysis (https://www.gtexportal.org/home/datasets). For analysis purpose to focus on the main tissues, various levels of the single tissue data were merged and presented as one (e.g. “sun” exposed and “no sun” exposed skin merged and presented as “skin” only). The downstream processing of read count data used for PCA analysis were done using edgeR software.^61^ To infer the kidney cell type compositions (deconvolution) from the bulk uEV mRNA profiling, we performed a deconvolution analysis using single-nucleus RNA sequencing data from Wu et al.^25^ We analysed the reference data using ScType pipeline and estimated the 11 kidney cell types using markers available at the ScType database.^27^ We then applied a single cell deconvolution algorithm using BisqueRNA^63^ for estimations of those kidney cell type proportions.

#### Taqman qPCR

Selected differentially expressed genes from mRNAseq were validated using Taqman qPCR in Fluidigm Biomark^TM^ HD 96.96 platform (Fluidigm, San Francisco, CA, USA) according to manufacturer's instructions. Briefly, 1,25 μl of cDNAs (see preparation from RNA sequencing) were preamplified with 14 PCR cycles according to the instructions in Fluidigm’s Real-Time PCR Analysis User Guide (PN 68000088 K1); the conditions were 95°C for 2 min, followed by 14 cycles of 95°C for 15 s and 60°C for 4 min. The number of preamplification cycles, 14 against 20, was tested separately with a part of the assays and samples, where 14 cycles gave better Ct values and amplification curves (data not shown). We applied one to two different assays for each gene, and 4-6 technical replicates, including two reverse transcription replicates, of each sample/assay. Water instead of sample served as a negative control. The assays with FAM-MGB labelled probes were: MAP7 (Hs01010631_m1, Hs01009609_m1), GPX3 (Hs00173566_m1, Hs01078668_m1), IL32 (Hs00170403_m1, Hs00992441_m1), NOX4 (Hs04980924_m1, Hs01379108_m1), HRSP12 (Hs01066170_m1), GAPDH (Hs99999905_m1) (all Applied Biosystems; Thermo Fisher Scientific, Inc., Waltham, MA, USA). The qPCR was performed using the online protocol “Gene Expression with the 96.96 IFC Using Fast TaqMan Assays (Biomark HD Only) (PN 100-2638 D1)” with conditions of 95°C for 60 s and 35 repeats of 97°C for 5 s and 60°C for 20 s. Data was analyzed with the method according to Livak and Schmittgen et al.^69^ with normalization using GAPDH. Samples with less than two successful replicas or high deltaCt (>19) were excluded from the analysis.

### Quantification and statistical analysis

#### Pathway analysis and statistics

Details of statistical analysis used for each section have been described in figure legends and manuscript text. Briefly, differential expression analysis was performed based on count data using generalized linear models, as implemented in edgeR software,^61^ adjusting for age, BMI, diabetes duration and urine collection protocols (ON and 24-h) or other covariates (described in Figure 4) as appropriate. Database for Annotation, Visualization and Integrated Discovery (DAVID) v6.8 was used for pathway analysis.^70^^,^^62^ For qPCR data analysis, deltaCt values were transformed using inverse normal transformation (Figure S6). Correlation analysis were performed using non-parametric Spearman’s correlation (two tailed, Figure 4). The transcriptional risk score was derived by averaging the inverse rank normalized expression values of the candidate genes (Figures 6 and 7). Slopes in the longitudinal data analysis were estimated using linear regression analysis (Figure 7). Data is presented as Mean ±SEM or box and whiskers plot (interquartile range, median and summary minimum/maximum values) as described in figures.

### Additional resources

The iBEAt study (Prognostic imaging biomarkers for diabetic kidney disease) has been registered as clinical trial study (Clinicaltrials.gov NCT03716401, Gooding et al.^58^).

## Data Availability

• This paper does not report original code. • The raw count data for all the genes from total mRNAseq used in manuscript (discovery phase - T1D FinnDiane and DIREVA studies and technical comparisons) are provided in the Supplementary excel data material (Tables S9, S10, and S11). In addition, we provide a web data browser for exploring the mRNA expression in the study groups (https://uev-mrna.rahtiapp.fi/). This resource is publicly available as of the date of publication. The raw individual-level FASTQ data from mRNAseq included in this study will be available for all researchers fulfilling the study consents. • Any additional information required to reanalyze the data reported in this paper is available from the lead contact upon request compatible with the study consents.

## Supplemental information

Document S1. Figures S1–S7 and Tables S1–S7

Table S8. Pathway enrichment analysis of the differentially expressed genes, related to Figure 6 and Table S7

Table S9. Raw gene count data of both T1D cohorts (n = 72 samples) used for differential expression analysis, related to Figure 4

Table S10. Raw gene count data of technical comparison samples, related to Figure 2

Table S11. Differential gene expression analysis results for all the genes, related to Figure 4A
