# Supplementary material for: Genome-wide mRNA profiling in urinary extracellular vesicles reveals stress gene signature for diabetic kidney disease
Source: iScience. 2023 Apr 18;26(5):106686. doi: 10.1016/j.isci.2023.106686 (PMC10193229; doi:10.1016/j.isci.2023.106686)
Supplement: Document S1. Figures S1–S7 and Tables S1–S7 [file mmc1.pdf]

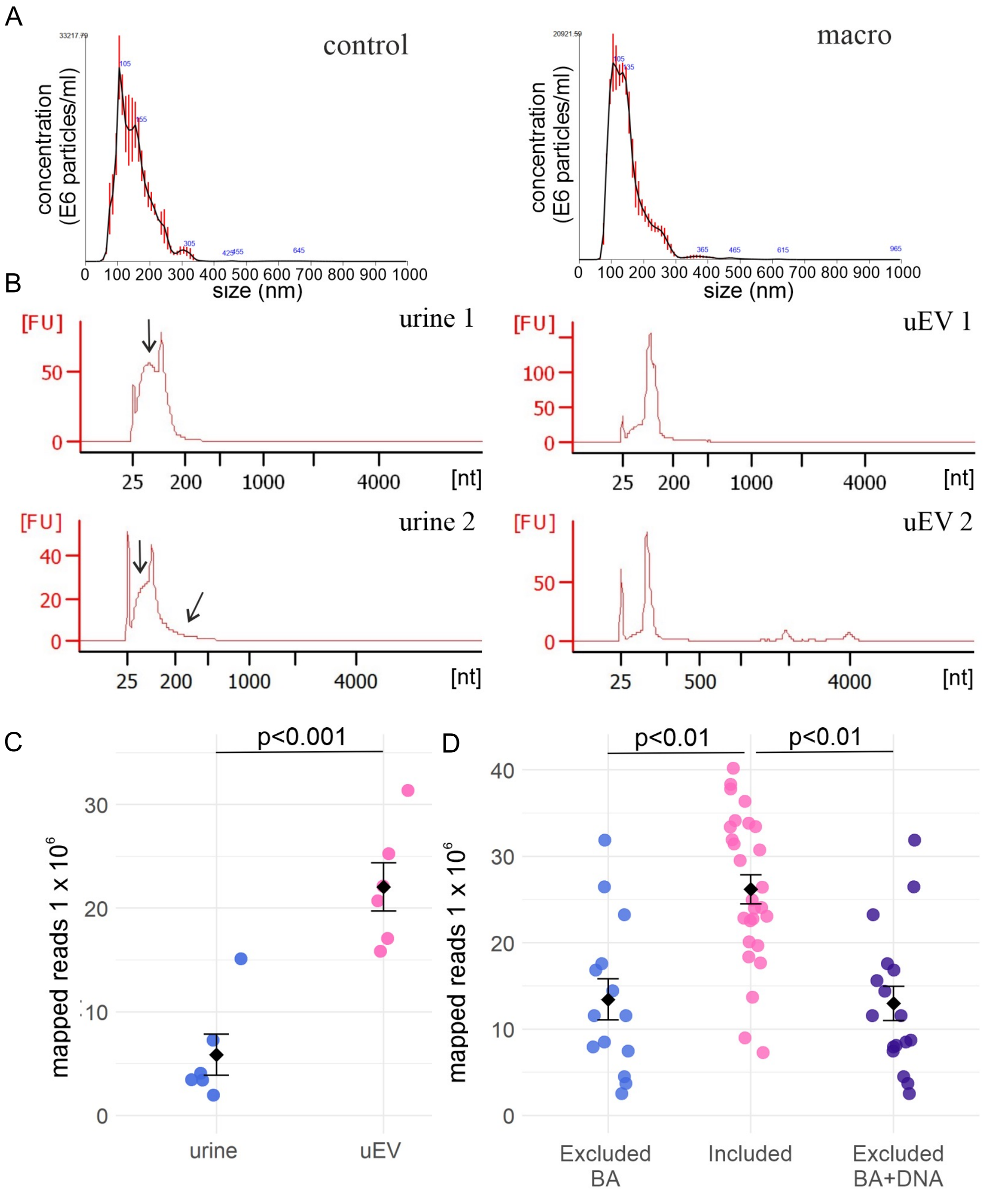

Supplementary Figure 1: Quality control of the uEV preparations (*Related to Figure 1*)

(**A**), Representative nanoparticle tracking analysis profiles showing enrichment of uEV sizes between 50-300 nm.
(**B**), Examples of Bioanalyzer Pico assay profiles of paired RNA samples extracted from whole urine and uEV of the same urine sample. The RNA profiles from urine showed characteristic signs of degradation (arrows) that were missing from uEV.
(**C**) Urine RNA samples gave a significantly reduced number of mapped reads in mRNAseq as compared to their paired uEV RNA samples (n=6 controls).
(**D**) Quality assessment of RNA in a subset of women's T1D cohort (n=44). Excluded uEV samples that showed signs of degradation in their bioanalyzer (BA) RNA profile gave a significantly reduced number of mapped mRNAseq reads compared to the included uEV samples without the signs. Similar results were obtained for all excluded samples using both BA RNA profile and potential DNA contamination (based on intergenic reads) as exclusion criteria.
Messenger RNA sequencing (mRNAseq), type 1 diabetes (T1D), urinary extracellular vesicles (uEV).

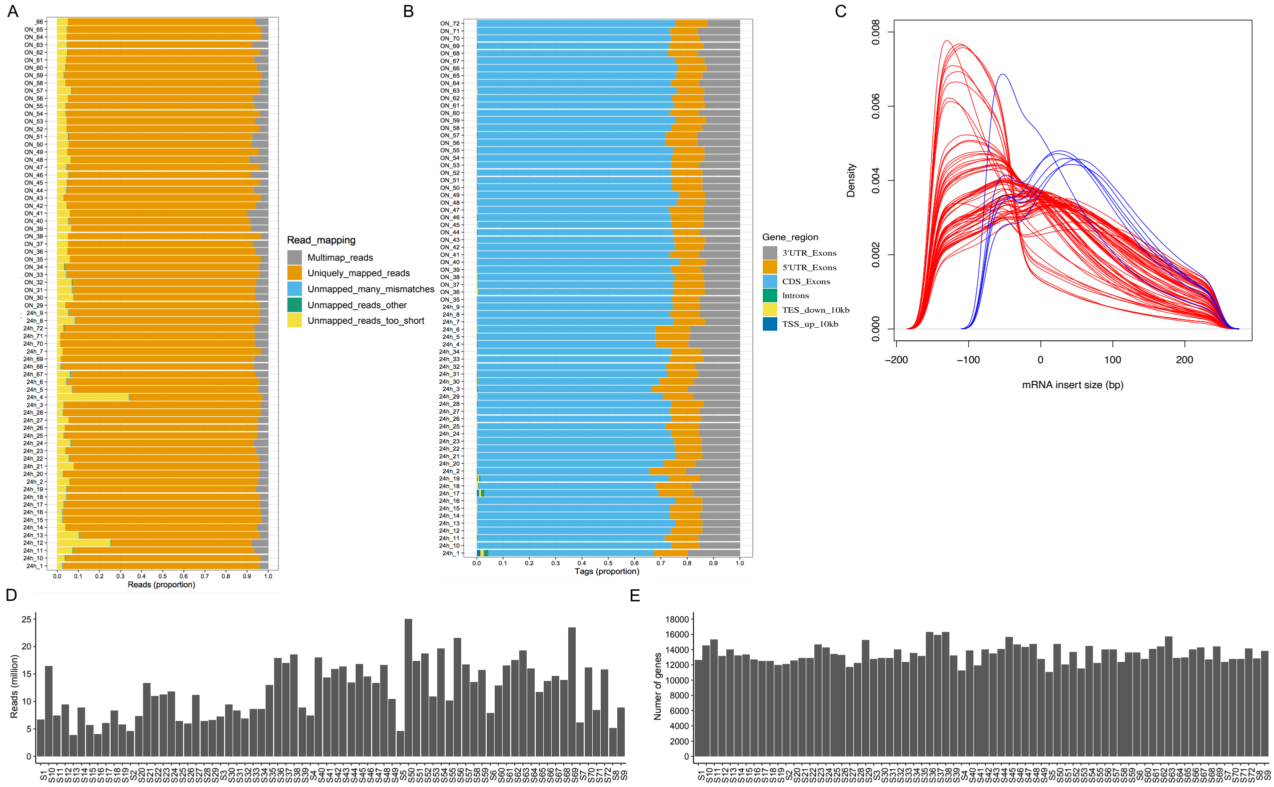

Supplementary Figure 2: Mapping of raw sequecing reads from uEV mRNA sequencing to human genome in the whole T1D discovery cohort (*Related to Figure 1)*

(A), Figure shows the proportions of uniquely-mapped, multi-mapped and unmapped uEV mRNA sequencing reads to human genome in each sample. **(B)**, Figure shows the proportions of tags mapping to exons, 3' or 5' UTR, introns or intergenic parts i.e. TSS or TES 10 kb (10 kb upstream of TSS or downstream of TES) in each sample. Alignments of the raw sequecing reads to human genome were done using STAR [S2].

(C), Distribution of insert sizes (inner distance) in RNA sequencing. Color coding is for two different lengths of paired end reads (blue=75bp and red=150bp) used for RNA sequencing in discovery phase.

(D and **E)**, Distribution of D- raw sequecing reads (paired end) after RNA sequecing and E- numbers of genes detected (with ≥1 count) per sample.

Coding sequence (CDS); overnight (ON); Type 1 diabetes (T1D), transcriptional end site (TES); transcriptional start site (TSS); urinary extracellular vesicles (uEV); untranslated region (UTR); 24 hour (24h).

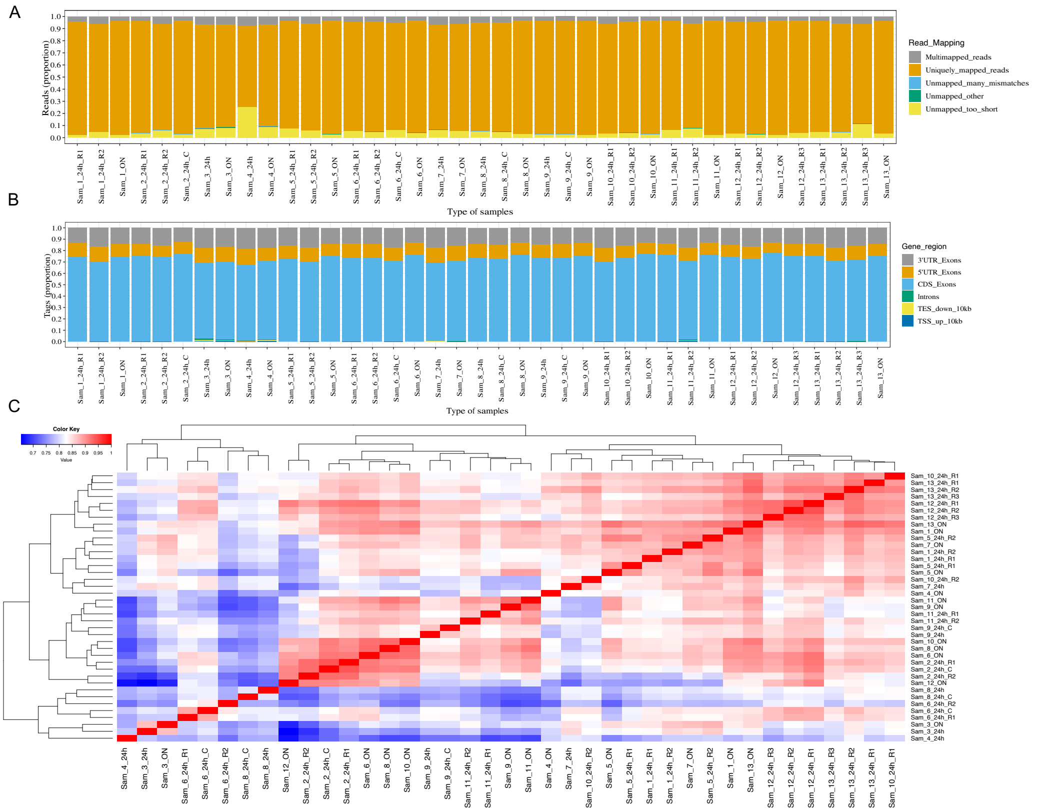

Supplementary Figure 3: Mapping distributions and correlation of the mRNA sequencing reads in the uEV samples used for technical comparisons of urine collection type, centrifugation and reproducibility (*Related to Figure 1)*

(A), Proportions of uniquely mapped, multi-mapped and unmapped raw sequencing reads.
(B), Proportions of tags mapping to exons, 3' or 5' UTR, and introns or intergenic parts i.e. TSS or TES 10 kb (10 kb upstream of TSS or downstream of TES).
(C), Sample-to-sample correlation heatmaps and hierarchical clustering based on count data (n= 10,449 genes). Technical sample comparisons included: 1) Technical replicates (_R); aliquots of individual samples (24h urine collection) were processed through the uEV isolation and mRNA sequencing pipeline twice (n=6 donors, T1D: Samples 1, 2, 5 and 6; Non-diabetic: Samples 10 and 11) or thrice (n=2 non-diabetic donors: Samples 12 and 13) at 1-5 months intervals. 2) Centrifugation test samples; aliquots of individual urine samples (24h collection) were processed with (_C) and without centrifugation before freezing (n=4 T1D donors: Samples 2, 6, 8 and 9). 3) Samples from ON vs 24h urine collections; samples from individual donors were collected from the same day (n=12 donors, T1D: Samples 1, 3-9; Non-diabetic: Samples 10-13). Correlation analysis was performed using non-parametric Spearman's (two-tailed) method.
The gene expression values (log2CPM) were inverse normally transformed and converted to Z score before analysis.
Counts per Million (CPM); overnight (ON); Sam (Samples); Type 1 diabetes (T1D), transcriptional end site (TES); transcriptional start site (TSS); urinary extracellular vesicles (uEV); untranslated region (UTR); 24 hour (24h). Data for 9 samples in (**A**) and **(B)** overlap with Figure 2 and S2, respectively, and have been used as a reference in their respective paired analysis: Sam_1_24h_R1, Sam_2_24h_R1, Sam_3_24h, Sam_4_24h, Sam_5_24h_R1, Sam_6_24h_R2, Sam_7_24h, Sam_8_24h, Sam_9_24h.

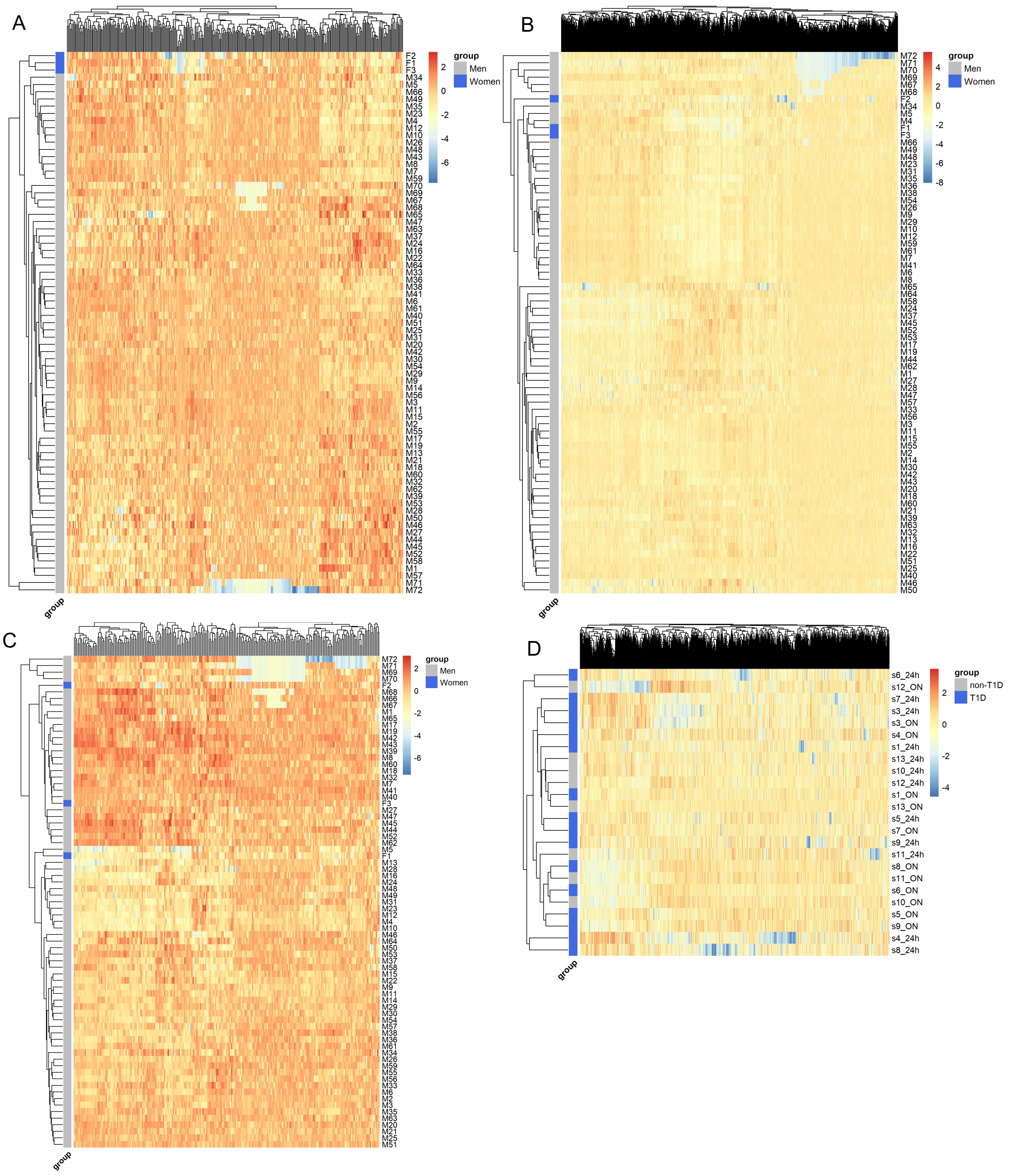

Supplementary Figure 4: Comparison of uEV mRNA profiles between men and women (*Related to Figure 1*) and technical replicates (*Related to Figure 2*)

Clustering based on the expression levels of **(A)** a subset of sex chromosome genes (chromosome X and Y, n=329 genes) or **(B)** whole uEV mRNA transcriptome (n=10, 596 genes) or
(C) a subset of kidney-enriched genes (n=247, based on Human protein Atlas, see Methods) for all samples. Analysis included three samples from female (F) donors (24h urine collection) and 72 samples from male (M) donors (24h and ON urine collections) of the T1D cohorts used in discovery phase.
(D), Clustering based on expression levels of all uEV genes (n=10,449) for technical replicate samples from ON vs 24h urine collections (T1D: Samples S1-S9 and S10-S13: non-diabetic, all from male donors) as shown in ***Figure 2***. Clustering analysis was performed using Euclidean distance based method.
T1D, Type 1 diabetes; uEV, urinary extracellular vesicles.

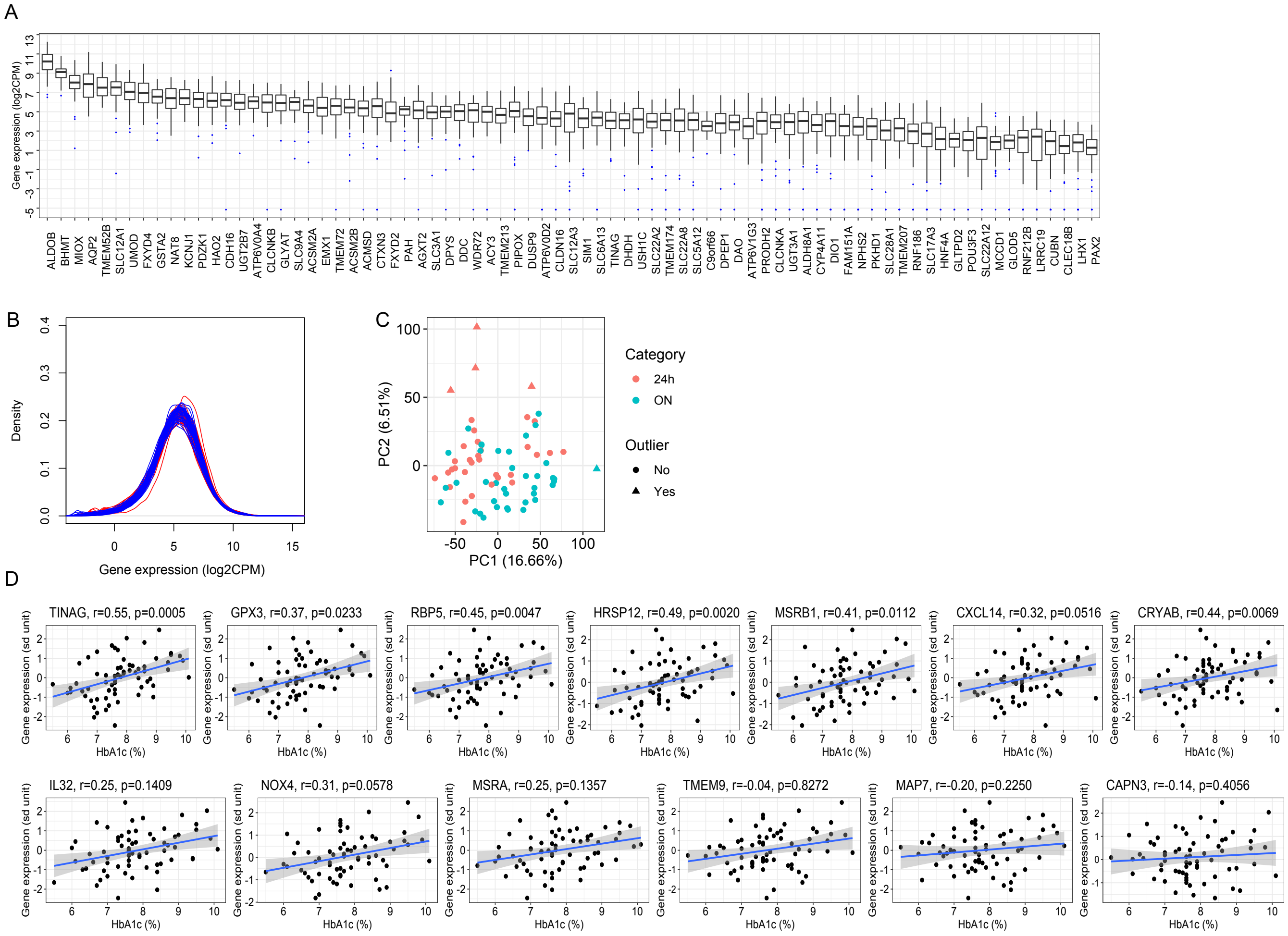

Supplementary Figure 5: Characterization of uEV mRNA profiles at discovery phase (*Related to; A- Figure 3, B to D- Figure 4*)

(**A**), The boxplot depicts the interquartile range, median and minimum/maximum summary values of expression levels for the 77 known specific kidney enriched genes that are reported to be detected in kidney and only in some other human tissues (see Methods).
(**B)**, Density plot depicting mRNA expression (log2CPM) distribution for all commonly expressed protein coding genes (count ≥1 in ≥90% samples, n=10,596) in uEV samples from the T1D discovery cohort (total n=72; 24h (24 hour) collections in red, n=34; ON (overnight) collections in blue, n=38). (**C)**, Principal component (PC) analysis using mRNA expression data for all commonly expressed genes (n=10,596) in the same samples as B.
(**D**), Correlation between expression level of the top 13 differentially expressed genes and HbA1c level (%) in normoalbuminuric individuals of the T1D discovery cohort (n=37). HbA1c was measured close to the time of urine collection. The gene expression values (log2CPM) were inverse normally transformed and converted to z score units (sd unit) before Spearman's correlation analysis (two tailed). Counts per Million (CPM).

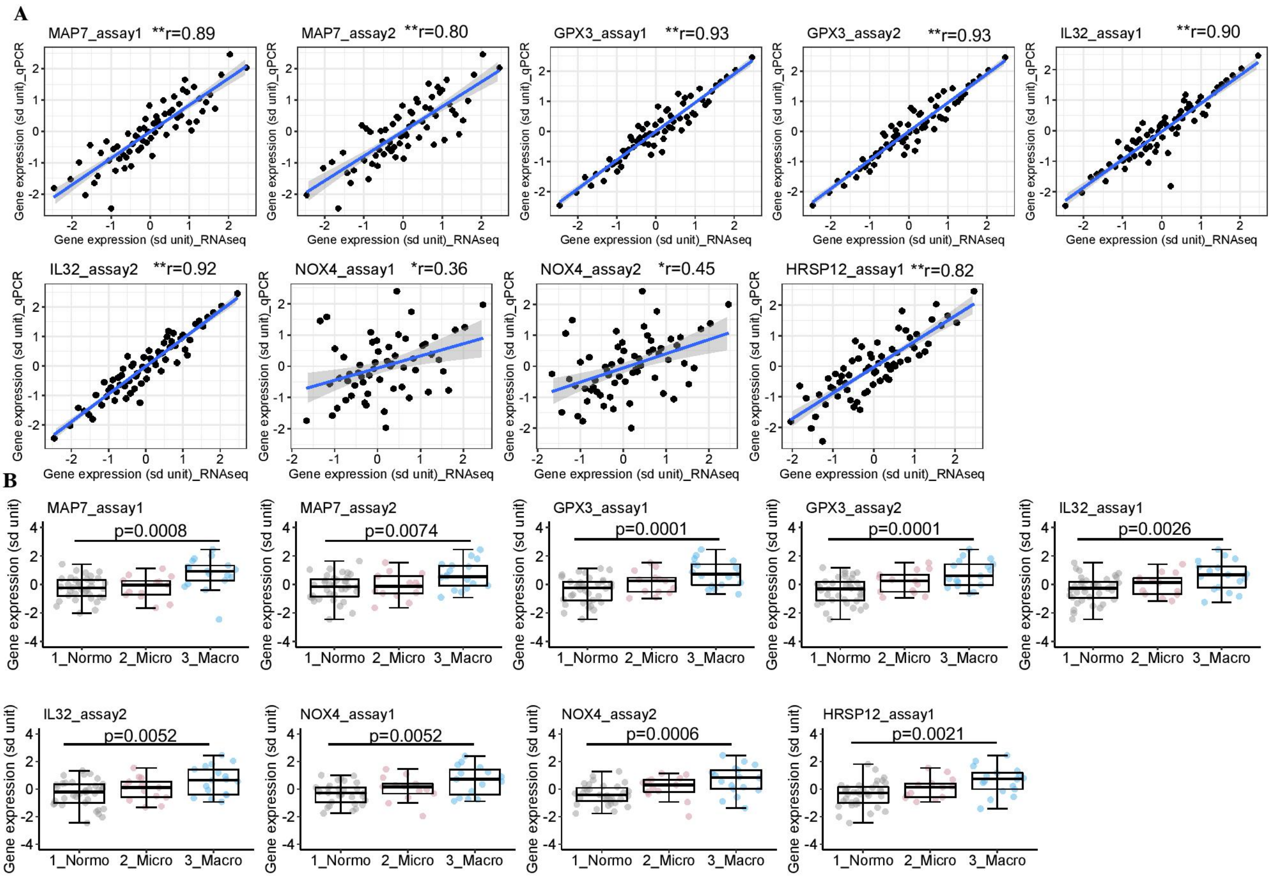

Supplementary Figure 6: Validation of mRNA sequencing -based differentially expressed genes using quantitative PCR (*Related to Figure 4*)

Differential expression of five of the top 13 DKD associated transcripts of uEV (*MAP7, GPX3, IL32, NOX4, HRSP12*; Fig. 5) was validated by qPCR in the T1D discovery cohort.

(**A)**, Correlation of the gene expression level by mRNA sequencing (x-axis) vs. qPCR (y-axis) in the same samples (n=57-66). DeltaCt values from the qPCR experiments and log2 counts per million values from sequencing experiments were inverse normally transformed and converted to z score unit (sd unit); r=non-parametric Spearman's (two-tailed) method; *p<0.006, **p<0.00001.

(**B)**, The boxplots (interquartile range, median and minimum/maximum summary values) show the gene expression level derived from qPCR assays using 1-2 Taqman assays (assay1 and assay 2) in the T1D cohort stratified for the degree of albuminuria (normo-, n=29-36, micro-, n=13, and macroalbuminuria, n=15-17). The gene expression values (deltaCt values from qPCR) were inverse normally transformed and converted to z score unit (sd unit) and statistical comparisons of the three groups were performed using one-way ANOVA.

Cycle threshold (Ct); Diabetic kidney disease (DKD); macroalbuminuria (Macro); microalbuminuria (Micro); normoalbuminuria (Normo); quantitative PCR (qPCR); type 1 diabetes (T1D); uEV; urinary extracellular vesicles (uEV).

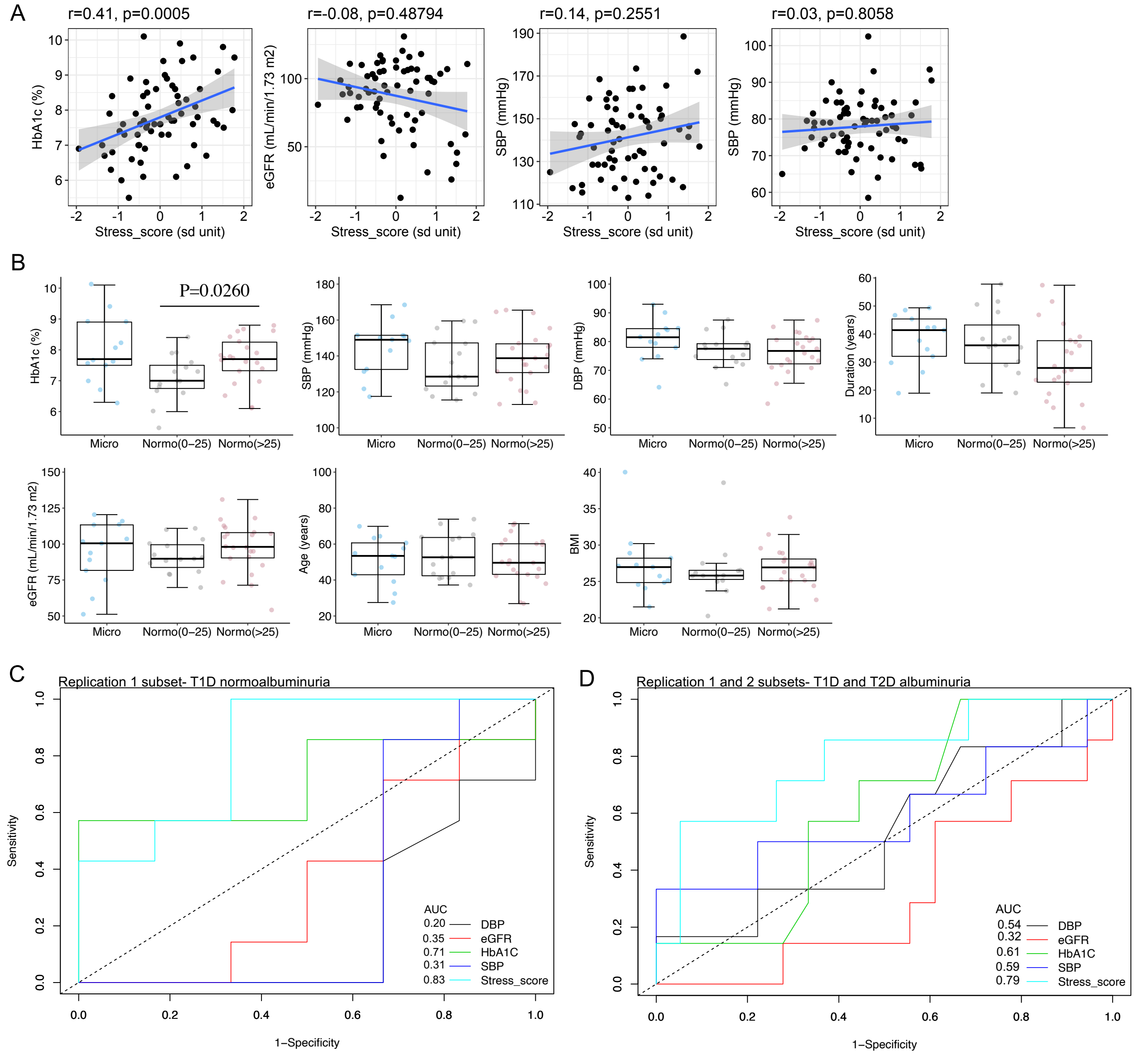

Supplementary Figure 7: Association of uEV stress score with clinical parameters (*Related to Figure 6 and 7*)

(A), Correlation of the transcriptomic stress scores in T1D discovery cohort (normo-, n=37, micro- n=13 and macroalbuminuria, n=17) with clinical parameters involved in modulation of kidney function. **(B)**, Distribution of clinical data measured at the time of uEV collection among the T1D discovery cohort including microalbuminuria group (n=13), and normoalbuminuria group stratified for the stress score (lowest quartile, n=15; two middle quartiles, n=22) as used in **Figure 7B**.
(C and **D)**, ROC (receiver operator characteristic) analysis of the stress score and other known clinical parameters in replication cohorts for predicting eGFR decline (mL/min/1.73 m2/year) of **C**- ≤-0.5 in T1D normoalbuminuria group (n=13 women, as shown in **Figure 7D**) and **D**- ≤-3.07 in combined T1D and T2D albuminuria groups **(**n=26, 8 women and 18 men, available for DIREVA and FinnDiane study; 26 out of 34, **Table S3**). The latter cutoff was based on lowest 25% percentile of eGFR slope distribution in all 26 individuals with DKD.

Table S1: **Clinical characteristics of the two type 1 diabetic cohorts (all male) used in the discovery phase of the study (*Related to Figure 1)***

**Total Numbers (Normo/Micro/Macro)**	**24-hour collection** 34 (13/7/14)		**Overnight collection** 38 (25/8/5)	
	n	Mean (SEM)	n	Mean (SEM)
Age (years)	30	50.3 (2.4)	38	51.8 (2.0)
BMI (kg/m^2)	30	27.9 (0.9)	36	26.1 (0.5)
WHR	30	0.96 (0.01)	38	0.95 (0.01)
HbA1c (%)	30	7.9 (0.2)	38	7.7 (0.2)
SBP (mmHg)	30	146 (3.3)	38	137 (2.5)
DBP (mmHg)	30	77 (1.6)	38	79 (1.1)
Diabetes duration (years)	30	34.0 (2.3)	38	36.2 (1.6)
eGFR (mL/min/1.73 m2)	30	81.5 (5.4)	38	91.6 (3.2)
*AER (mg/24 hours)	25	300.7 (89.7)	--	--
$^{\#}$AER (µg/min)	--	--	37	38.4 (11.7)

Normo=normoalbuminuria; Micro=microalbuminuria; Macro=macroalbuminuria; WHR=waist-to-hip ratio; HbA1c= glycated hemoglobin; SBP=systolic blood pressure; DBP= diastolic blood pressure; eGFR=estimated glomerular filtration rate; *AER= albumin excretion rate (mg/24 hours); $^{\#}$AER= albumin excretion rate (µg/min); n=total numbers.

Table S2: Clinical characteristics of the type 1 diabetic discovery cohorts (all male) stratified into normo-, micro- and macroalbuminuria groups that were used in global differential expression analysis (*Related to Figure 4*)

Total Numbers	Normo 37		Micro 13		Macro 17		p value
	n	Mean (SEM)	n	Mean (SEM)	n	Mean (SEM)	
Age (years)	37	51 (2.2)	13	51.3 (3.6)	17	50.5 (2.8)	0.9720
BMI (kg/m^2)	35	26.7 (0.6)	13	27.3 (1.2)	17	27.7 (1.2)	0.8370
WHR	35	0.95 (0.01)	13	0.93 (0.02)	17	0.99 (0.02)	0.0394
HbA1c (%)	37	7.4 (0.1)	13	8.0 (0.30)	17	8.5 (0.2)	0.0060
SBP (mmHg)	37	137.6 (2.5)	13	144.5 (4.1)	17	147.2 (4.9)	0.1408
DBP (mmHg)	37	76.6 (1.1)	13	80.8 (2.0)	17	78.8 (2.4)	0.1771
Diabetes duration (years)	37	32.9 (2.1)	13	38.1 (2.6)	17	37.2 (1.9)	0.2016
eGFR (mL/min/1.73 m2)	37	95.2 (2.6)	13	94.0 (6.0)	17	65.3 (7.6)	0.0041
*AER (mg/24 hours)	13	16.3 (4.4)	3	101.9 (87.7)	9	777.7 (147.1)	0.0002
#AER (μg/min)	24	5.6 (1.5)	8	57.3 (12.4)	4	206.7 (48.6)	<0.0001
Medication (%)		46		85		94	

P values were calculated using Kruskal-Wallis statistics. Medication data are for ACE inhibitors and/or Beta blockers.
Normo=normoalbuminuria; Micro=microalbuminuria; Macro=macroalbuminuria; WHR=waist-to-hip ratio; HbA1c= glycated hemoglobin; SBP=systolic blood pressure; DBP= diastolic blood pressure; eGFR=estimated glomerular filtration rate; *AER= albumin excretion rate (mg/24 hours);
#AER= albumin excretion rate (μg/min); n=total numbers.

Table S3: Clinical characteristics of the diabetic cohorts used in replication studies and stratified according to albuminuria status (*Related to Figure 6 and 7*)

	**DIREVA T2D study**		**FinnDiane T1D study**		**iBEAt T2D study**	
Total numbers (male/female)	Micro 14 (12/2)	Macro 8 (7/1)	Normo (0/18)	Micro & Macro (0/12)	A1 (1/8)	A2&A3 (11/3)
Age (years)	69.2 (1.23)	71.58 (2.59)	44.55 (2.15)	46.60 (3.49)	66.75 (3.61)	65.64 (3.48)
BMI (kg/m^2)	30.91 (1.13)	33.46 (1.55)	25.50 (0.94)	29.97 (2.38)	32.61 (1.47)	29.88 (0.87)
WHR	1.07 (0.02)	1.07 (0.03)	0.81 (0.016)	0.87 (0.021)	0.96 (0.025)	1.04 (0.016)
HbA1c (%)	7.74 (0.44)	8.31 (0.44)	7.66 (0.20)	7.86 (0.22)	--	--
SBP (mmHg)	138.29 (4.46)	151.36 (10.98)	121.38 (5.44)	126.06 (4.43)	140.57 (9.30)	154.80 (6.77)
DBP (mmHg)	81.96 (2.35)	79.07 (3.99)	77.68 (2.38)	75.50 (3.06)	80.94 (5.22)	86.09 (3.34)
Diabetes duration (years)	19.73 (2.07)	18.73 (2.22)	31.90 (2.81)	34.76 (3.27)	10.87 (2.12)	19.07 (2.17)
eGFR (mL/min/1.73 m2)	63.46 (4.89)	61.61 (7.02)	104.49 (3.99)	73.55 (8.78)	--	--
#AER (µg/min)	125.9 (27.37)	>350*	--	--	--	--
AER (mg/24 hours)	--	--	9.27 (0.88)	156.20 (121.07)	--	--
Medication (%)	100	100	37.5	75	--	--

Data are mean (sem) or percentage. Medication data are for ACE inhibitors and/or Beta blockers. A1-3 according to KDIGO (see methods).
Micro=microalbuminuria; Macro=macroalbuminuria; Normo=normoalbuminuria; WHR=waist-to-hip ratio; HbA1c= glycated hemoglobin; SBP=systolic blood pressure; DBP= diastolic blood pressure; eGFR=estimated glomerular filtration rate; ACR=Urine albumin-to-creatinine ratio at baseline visit.
#AER= albumin excretion rate (µg/min). *All clinically confirmed T2D macroalbuminuria subjects have AER >350 µg/min at the time of collection but their exact values were missing given that they all have raw urine albumin values above the upper detection limit of the test (>150). The AER data (24 hour or overnight urine collection based) was not available for iBEAt study [S1].

Table S4: Clinical characteristics of the T1D discovery cohorts (all male) stratified into groups based on chronic kidney disease (CKD) status (*Related to Figure 4*)

**Total Numbers**<br>**Normo/Micro/Macro**	**CKD stage ≤2**<br>46<br>33/9/4		**CKD stage ≥3**<br>20<br>3/4/13		**p value**
	n	Mean (SEM)	n	Mean (SEM)	
Age (years)	46	49.4 (2.0)	20	54.0 (2.4)	0.1698
BMI (kg/m^2)	46	26.9 (0.5)	18	27.6 (1.3)	0.9587
WHR	46	0.95 (0.01)	18	0.98 (0.02)	0.105
HbA1c (%)	46	7.6 (0.1)	20	8.4 (0.3)	0.0096
SBP (mmHg)	46	138.5 (2.4)	20	147.1 (3.9)	0.0768
DBP (mmHg)	46	78.5 (1.1)	20	77.1 (1.8)	0.5216
Diabetes duration (years)	46	32.1 (1.6)	20	40.9 (1.7)	0.0019
eGFR (mL/min/1.73 m2)	46	99.1 (2.0)	20	61.1 (5.7)	<0.0001
*AER (mg/24 hours)	15	148.3 (103.1)	9	585.4 (141.3)	0.0035
$^{\#}$AER (μg/min)	28	20.4 (6.9)	8	106.0 (42.0)	0.0214

The T1D individuals (66 out of 67) were classified into chronic kidney disease stages based on eGFR data including at least > 5 years follow up (see Methods for further details of classification). The table presents the mean values of the available clinical data measured at the time of urinary EV collection. P values were calculated using Mann-Whitney test.
CKD= chronic kidney disease; Normo=normoalbuminuria; Micro=microalbuminuria; Macro=macroalbuminuria; WHR=waist-to-hip ratio; HbA1c=glycated hemoglobin; SBP=systolic blood pressure; DBP= diastolic blood pressure; eGFR=estimated glomerular filtration rate; *AER= albumin excretion rate (mg/24 hours); $^{\#}$AER= albumin excretion rate (μg/min); n=total numbers; T1D= type 1 diabetes.

Table S5: Basic clinical characteristics of the donors of the samples used in technical comparison analysis of urine collection type, centrifugation and reproducibility (*Related to Figure 2*)

Sample_ID 1	Sample_ID 1	Sample_diabetes_status	Urine_albuminuria_status	Gender
Sam_1	s1	Type 1 diabetes	Microalbuminuria	Male
Sam_2	s2	Type 1 diabetes	Macroalbuminuria	Male
Sam_3	s3	Type 1 diabetes	Macroalbuminuria	Male
Sam_4	s4	Type 1 diabetes	Macroalbuminuria	Male
Sam_5	s5	Type 1 diabetes	Microalbuminuria	Male
Sam_6	s6	Type 1 diabetes	Macroalbuminuria	Male
Sam_7	s7	Type 1 diabetes	Macroalbuminuria	Male
Sam_8	s8	Type 1 diabetes	Microalbuminuria	Male
Sam_9	s9	Type 1 diabetes	Microalbuminuria	Male
Sam_10	s10	No diabetes	Normoalbuminuria	Male
Sam_11	s11	No diabetes	Normoalbuminuria	Male
Sam_12	s12	No diabetes	Normoalbuminuria	Male
Sam_13	s13	No diabetes	Normoalbuminuria	Male

Table S6: Comparison of the expression levels of the top differentially expressed genes between various stratified T1D groups of the discovery cohorts (*Related to Figure 4*)

**Total Numbers**	**Normo vs Micro**<br>37 vs 13		**Normo vs Micro & Macro**<br>37 vs 30		**CKD stage ≤2 vs CKD stage ≥3**<br>46 vs 20	
	logFC	p	logFC	p	logFC	p
MAP7	0.12	0.4119	1.14	$9.61{\times}10^{-9}$	0.91	$3.64{\times}10^{-5}$
MSRB1	0.28	0.1006	0.65	$1.09{\times}10^{-5}$	0.62	$8.67{\times}10^{-5}$
GPX3	0.49	0.1242	1.22	$3.29{\times}10^{-6}$	1.13	$5.30{\times}10^{-5}$
IL32	0.59	0.0646	1.28	$3.87{\times}10^{-6}$	1.12	$1.24{\times}10^{-4}$
TINAG	0.83	0.0352	1.34	$7.79{\times}10^{-6}$	0.97	$1.87{\times}10^{-3}$
NOX4	0.57	0.1134	1.45	$1.43{\times}10^{-6}$	1.37	$1.64{\times}10^{-5}$
HRSP12	0.23	0.2455	0.64	$2.62{\times}10^{-4}$	0.56	$2.35{\times}10^{-3}$
CAPN3	0.84	0.0961	1.69	$1.58{\times}10^{-5}$	1.47	$2.27{\times}10^{-4}$
CXCL14	0.38	0.2087	0.98	$6.68{\times}10^{-5}$	0.81	$1.65{\times}10^{-3}$
MSRA	0.59	0.0401	0.97	$4.16{\times}10^{-5}$	0.72	$3.56{\times}10^{-3}$
CRYAB	0.34	0.2293	0.90	$1.4{\times}10^{-4}$	0.88	$3.15{\times}10^{-4}$
RBP5	0.34	0.3034	1.02	$1.22{\times}10^{-4}$	0.95	$5.84{\times}10^{-4}$
TMEM9	0.49	0.0034	0.65	$6.01{\times}10^{-6}$	0.54	$5.57{\times}10^{-4}$

The two T1D cohorts were stratified based on albuminuria and chronic kidney disease stages based on eGFR data as shown in supplementary table **S2** and **S4** respectively. In the table, we present comparisons of the top 13 differentially expressed genes (obtained from Normo vs Macro comparison, as shown in **Figure 4**, between the T1D groups as indicated).
The differential gene expression analysis was performed based on count data using generalized linear models adjusting for age, body mass index, diabetes duration and urine collection protocols (overnight and 24 hour). Fold change is presented in log10 FC scale.
CKD= chronic kidney disease; FC, fold change; eGFR=estimated glomerular filtration rate; Normo=normoalbuminuria; Micro=microalbuminuria; Macro=macroalbuminuria; T1D= type 1 diabetes.

Table S7: Stress response related functions and association to kidney diseases for the proteins coded by the differentially expressed uEV transcripts used in the construction of the stress score (*Related to Figure 6 and 7*)

Stress score gene	Name	Biological funtion of proteins related to stress response , Reference	Association with the kidney diseases strength: -log10 (p-value)
CRYAB	Crystallin Alpha B	Chaperone function<br>Response to hydrogen peroxide and hypoxia<br>https://www.uniprot.org/uniprot/P02511	2.5
GPX3	Glutathione Peroxidase-3	Glutathione peroxidase activity<br>Cellular response to oxidative stress<br>Hydrogen peroxide catabolic process<br>Response to lipid hydroperoxide<br>Selenoprotein<br>https://www.uniprot.org/uniprot/P22352	2.5
NOX4	NAD(P)H oxidase 4	Oxygen sensor activity<br>Superoxide-generating NAD(P)H oxidase activity<br>Cellular response to oxidative stress<br>Reactive oxygen species metabolic process<br>Response to hypoxia<br>https://www.uniprot.org/uniprot/Q9NPH5	2.5
MSRB1	Methionine Sulfoxide Reductase B1	Peptide-methionine (R)-S-oxide reductase activity during Oxidative stress.<br>Protein repair<br>Selenoprotein<br>https://www.uniprot.org/uniprot/Q9NZV6	1.8
MSRA	Methionine Sulfoxide Reductase	Oxidoreductase<br>Protein repair<br>Cellular response to oxidative stress<br>https://www.uniprot.org/uniprot/Q9UJ68	1.8
HRSP12	Heat-responsive protein 12	Hydrolase<br>2-iminobutanoate deaminase activity<br>May facilitate the release of ammonia from potentially toxic reactive metabolites, reducing their impact on cellular components.<br>https://www.uniprot.org/uniprot/P52758	1.6

The information of molecular function and biological pathway related to stress responses were extracted from UniProt protein data base (http://www.uniprot.org). The information regarding association with kidney diseases were obtained from the Harmonizome database (https://maayanlab.cloud/Harmonizome/gene_set/Kidney+Diseases/CTD+Gene-Disease+Associations) (https://doi.org/10.1093/database/baw100).
In the table, the stress score candidate genes were ordered based on their association strength with the kidney diseases in the Harmonizome database. uEV, urinary extracellular vesicles.

References:

1. Gooding, K.M., Lienczewski, C., Papale, M., Koivuviita, N., Maziarz, M., Dutius Andersson, A.M., Sharma, K., Pontrelli, P., Garcia Hernandez, A., Bailey, J. et al. (2020). Prognostic imaging biomarkers for diabetic kidney disease (iBEAt): study protocol. BMC Nephrol. **21**(1), 242.
2. Dobin, A., Davis, C.A., Schlesinger, F., Drenkow, J., Zaleski, C,. Jha, S., Batut, P., Chaisson, M., Gingeras, T.R. (2013). STAR: ultrafast universal RNA-seq aligner. Bioinformatics **29**, 15-21.
